# WHO Environmental Noise Guidelines for the European Region: A Systematic Review on Environmental Noise and Permanent Hearing Loss and Tinnitus

**DOI:** 10.3390/ijerph14101139

**Published:** 2017-09-27

**Authors:** Mariola Śliwińska-Kowalska, Kamil Zaborowski

**Affiliations:** 1 Clinic of Audiology and Phoniatrics, Nofer Institute of Occupational Medicine, 8 Sw.Teresy Str., 91-348 Lodz, Poland; 2 Department of Physical Hazards, Nofer Institute of Occupational Medicine, 8 Sw.Teresy Str., 91-348 Lodz, Poland; kamil.zaborowski@imp.lodz.pl

**Keywords:** personal listening devices (PLD), equivalent sound pressure level, pure-tone audiometry (PTA), odds ratios

## Abstract

*Background*: Hearing loss is defined as worsening of hearing acuity and is usually expressed as an increase in the hearing threshold. Tinnitus, defined as “ringing in the ear”, is a common and often disturbing accompaniment of hearing loss. Hearing loss and environmental exposures to noise are increasingly recognized health problems. *Objectives*: The objective was to assess whether the exposure-response relationship can be established between exposures to non-occupational noise and permanent hearing outcomes such as permanent hearing loss and tinnitus. *Methods: Information sources*: Computer searches of all accessible medical and other databases (PubMed, Web of Science, Scopus) were performed and complemented with manual searches. The search was not limited to a particular time span, except for the effects of personal listening devices (PLDs). The latter was limited to the years 2008–June 2015, since previous knowledge was summarized by SCENIHR descriptive systematic review published in 2008. *Study eligibility criteria:* The inclusion criteria were as follows: the exposure to noise was measured in sound pressure levels (SPLs) and expressed in individual equivalent decibel values (L_EX,8h_), the studies included both exposed and reference groups, the outcome was a permanent health effect, i.e., permanent hearing loss assessed with pure-tone audiometry and/or permanent tinnitus assessed with a questionnaire. The eligibility criteria were evaluated by two independent reviewers. *Study appraisal and synthesis methods:* The risk of bias was assessed for all of the papers using a template for assessment of quality and the risk of bias. The GRADE (grading of recommendations assessment, development, and evaluation) approach was used to assess the overall quality of evidence. Meta-analysis was not possible due to methodological heterogeneity of included studies and the inadequacy of data. *Results:* Out of 220 references identified, five studies fulfilled the inclusion criteria. All of them were related to the use of PLDs and comprised in total of 1551 teenagers and young adults. Three studies used hearing loss as the outcome and three tinnitus. There was a positive correlation between noise level and hearing loss either at standard or extended high frequencies in all three of the studies on hearing loss. In one study, there was also a positive correlation between the duration of PLD use and hearing loss. There was no association between prolonged listening to loud music through PLDs and tinnitus or the results were contradictory. All of the evidence was of low quality. *Limitations:* The studies are cross-sectional. No study provides odds ratios of hearing loss by the level of exposure to noise. *Conclusions:* While using very strict inclusion criteria, there is low quality GRADE evidence that prolonged listening to loud music through PLDs increases the risk of hearing loss and results in worsening standard frequency audiometric thresholds. However, specific threshold analyses focused on stratifying risk according to clearly defined levels of exposure are missing. Future studies are needed to provide actionable guidance for PLDs users. No studies fulfilling the inclusion criteria related to other isolated or combined exposures to environmental noise were identified.

## 1. Introduction

According to the World Health Organization (WHO), approximately 5% (or 360 million) of world’ population has a disabling hearing impairment. Exposure to excessive noise is one of the major causes of hearing problems [1].

While the hazards to hearing stemming from exposures to occupational noise are well recognized and appropriate prevention programs have been developed, the risk of hearing loss related to environmental noise or sounds in general population is still not fully recognized.

Exposure to excessive noise is associated with damage to the sensory cells of the inner ear (cochlea), predominantly to the outer hair cells [2]. Oxidative stress and synaptic excitotoxicity are the major mechanisms of morphological pathologies [3]. Functional consequences of noise-induced cochlear damage comprise of a permanent hearing threshold shift, as well as poor speech-in-noise intelligibility. In humans, an early sign of noise-induced hearing loss (NIHL) is an audiometric notch at the frequencies 4–6 kHz. A hearing threshold shift related to intense noise exposure is occasionally temporary in its initial stages and is manifested as Temporary Threshold Shift (TTS). TTS may become permanent (Permanent Threshold Shift—PTS), due to repeated, chronic noise exposure, and/or due to a single exposure to intense noise. In addition, hearing loss due to noise exposure predisposes to the occurrence of tinnitus as a consequence of alterations in central auditory signaling [4]. Tinnitus can result in a decrease of concentration, the inability to relax, anxiety, and depression.

In view of the latest observation in animal models, overexposure to noise may also result in cochlear neuropathy, i.e., the progressive degeneration of auditory nerve fibers, which can develop without permanent damage to the outer hair cells (so called “hidden” hearing loss) [5]. Since “hidden” hearing loss is a phenomenon related to supra-threshold processes, in humans it may manifest as difficulties in understanding speech, in particular in noisy environments, and not as an audiometric threshold shift [6]. Noise is defined as all sounds that are undesirable or unpleasant, as well as harmful acoustic air vibrations perceived by the ear or other parts of human body. It is worthwhile to note that several exposures to environmental sounds (noises) do not fall under this definition since they are intentionally produced and listened to for pleasure (e.g., music). However, still, excessive exposure to such sounds may produce hearing loss.

The intensity of sounds (noise) is measured in logarithmic decibel (dB) units. The decibel is defined as the logarithm of the ratio between the value of the acoustic pressure of a given sound, measured in Pascals (Pa), and the value of 20 µP, which is the perception threshold (i.e., the lowest sound pressure level perceived by human ear). In the logarithmic unit scale, simple addition is not possible. Therefore, a formula of 3 dB exchange rate is commonly used, which means that doubling in sound energy equates to about 3 dB increase of sound pressure level. Major factors determining the development of NIHL are: the intensity of noise (measured in sound pressure levels, SPLs, usually with an A-weighted filter), length of exposure (expressed in years), impulsiveness of noise (impulsive noise is more dangerous than continuous noise; the intensity of impulse noise is measured with an C-weighted filter), and individual susceptibility for developing NIHL. The A-weighting is an internationally standardized characteristic in which sensitivity varies with frequency in the same way as the human ear, thus simulating the equal loudness contours. The A-weighting modifies the frequency response to follow approximately the equal loudness curve of 40 phons (high-pass filter). Since the levels of noise vary over time, the level of exposure for assessing the risk of NIHL in occupational setting is calculated as an A-weighted equivalent sound pressure level, averaged over the time of an 8-h working day (L_EX,8h_), or 40 h of working week (L_EX,week_).

The Directive 2003/10/EC of the European Parliament and the Council of 6 February 2003, on the minimum health and safety requirements regarding the exposure of workers to the risks arising from physical agents (noise), constitutes the current European legislation regulating the protection of workers from the risk of NIHL [7]. Based on the ISO 1999 standard [8], the Directive sets limits of exposure depending on the equivalent noise level for an 8 h working day and obliges the employer to take suitable steps if the limits are exceeded. The Noise at Work Regulation [7] recommends three action levels for occupational settings:

- the first (lower) action level set at L_EX,8h_ = 80 dBA;
- the second (upper) action level set at L_EX,8h_ = 85 dBA; and,
- the maximum exposure limits set at L_EX,8h_ = 87 dBA.

The limits are to be converted using the time-intensity trade-off of 3 dB increase for halving the time. For example, the exposure to noise at 97 dBA for 30 min a day would equate to the second action level (85 dBA for 8 h), under the assumption of long term exposure (Table 1). The first, lower action level of 80 dBA is the most conservative value, below which no consequences of exposure to occupational noise are expected.

To date, no commonly accepted method for assessing the risk of hearing loss due to environmental exposures to noise has been developed. Thus, the ISO standard and the Directive 2003/10/EC are the main tools used for these purposes.

### 1.1. Leisure Activities

There are several types of leisure activities accompanied by loud sounds, such as attending nightclubs, pubs, and fitness classes; live sporting events; concerts or live music venues; and, listening to loud music through personal listening devices (PLDs). Among these, the nightclubs were proven to be the main source of high-risk leisure noise [9]. Exposure levels in these venues were measured and referred to by survey data of 1000 Australian young adults. Using population weighted metrics the majority of study participants had exposures within the acceptable workplace noise limit as referred to the action level of L_Aeq8h_ = 85 dB. However, 14% were overexposed and a correlation between the increase of exposure to leisure-noise experienced and tinnitus was observed. Alarming data about health effects comes also from a recent study completed in over 300 British students [10]. As many as 88% of subjects experienced temporary tinnitus after leaving a nightclub, and 66% suffered temporarily impaired hearing the following morning. Over 70% felt that the noise at the nightclub should be limited to safe levels, however a similar percentage of study subjects claimed that they would attend clubs despite the risk of hearing loss.

The risk factors for NIHL related to different kinds of noise were further characterized for over 4500 inhabitants of New York City by collecting survey data. It was shown that the highest risk for hearing loss at 4000 Hz was associated with high occupational exposure, however a greater number of individuals were at risk of NIHL from MP3 players and stereos [11]. This was true even though, on average, the time of their use represents only a small fraction of total annual hours for each subject. Similarly, the study on over 8700 adolescent females of lower socioexconomic status showed that the use of PLDs was highly correlated with high frequency (3000, 4000, and 6000 Hz) hearing impairment and tinnitus, although this relationship was independent from time of the PLDs use. The authors suggest that PLDs might be the most important cause of doubling of hearing loss in this subgroup of the society over the last 24-year period [12]. The association between slight-mild acquired sensorineural hearing loss and the parent report of personal stereo use has also been shown in the large study on over 6500 elementary school children in Australia, making the safety of listening music through the PLDs a major public health problem [13].

Nowadays, as many as 88 to 90% of teenagers and young adults report listening to music through PLDs earphones [14,15]. The WHO estimated that 1.1 billion young people worldwide could be at risk of hearing loss due to unsafe listening practices [16]. Since NIHL develops very slowly (over the years of exposure), a large body of the most recent literature was aimed at predicting how many teenagers and young adolescents would develop permanent hearing loss in the future as a consequence of listening to the music through PLDs. 

Two systematic reviews have summarized recent estimates of the risk of developing permanent hearing loss due to the use of PLDs. The first one is the report of the Scientific Committee on Emerging and Newly Identified Hazards and Risk (SCENIHR), which was delivered by a group of experts on 23 September 2008 [17]. The report concludes that prolonged exposure to sounds from PLDs may possibly result in: temporary hearing threshold shift (TTS), permanent hearing threshold shift (PTS), and tinnitus, as well as poor speech communication in noisy conditions. Referring the levels and times of exposure to the first action level of ”Noise at Work Regulations”, it was estimated that approximately 5–10% of the listeners are at risk of developing permanent hearing loss after five or more years of exposure. The risk of hearing loss can be higher when using insert earphones when compared with headphones and can depend on the type of music and listening environments (the level of background noise). However, based on the data available by 2008, there was no direct evidence for an effect of repeated, regular daily exposures to music listened to through PLDs on the development of permanent NIHL. Data on tinnitus was inadequate and therefore non-conclusive. For none of the hearing effects, a meta-analysis was performed, nor were the exposure-effect curves reported. The SCENIHR report was based on a narrative review of 30 original papers with over in total about 2000 participants and exposures to music sounds that covered a range of 60–120 dB. Studies included in this review were carried out between 1982 and 2007.

Subsequent to the SCENIHR report of year 2008, several individual studies were published, aimed at predicting the risk of permanent hearing loss and tinnitus in a given population from PLDs usage. The studies were done e.g., in American, Australian, Austrian, Canadian, Dutch, Italian, Jewish, Chilean, Brazilian, and Malaysian teenagers and young adults [14,15,18,19,20,21,22,23,24,25].

Depending on the standard of risk assessment and the background noise, the percentage of the population that could be affected by noise damage is within the range of 0–29% (Table 2). Using the most common reference (safety limit) value of L_Aeq8h_ > 85 dBA (Directive 2003/10/EC—second action level, NIOSH criteria [26]), 3.2% to 26% of individuals were estimated to exceed this level limit [18,19,22,23,27]. By using lower reference limit of L_Aeq8h_ > 80 dBA (first action level) the percent of individuals at risk increased to almost 29% [14].

Very recently, in 2014, the second systematic review entitled “Personally modifiable risk factors associated with pediatric hearing loss” has been published [28]. Although the objective of this publication was to determine threshold levels of personally modified risk factors for hearing loss in the pediatric population, specific thresholds analyses were limited. Based on descriptive overview of original papers, the authors identified exposures to loud music (including the usage of PLDs) and working on a mechanized farm as the main risk factors for hearing loss in children and teenagers. Thresholds of exposures to music significantly associated with hearing loss in youth were as follows: (1) more than 4 h per week or more than 5 years of personal headphone usage; (2) more than 4 visits per month in a discotheque.

### 1.2. Aircraft, Road and Rail Noises

Some older studies (published over 20–30 years ago) suggested that living or attending a school close to an airport was associated with pediatric hearing loss [29,30]. One cohort study demonstrated a significantly worse standard pure-tone average, high pure tone average, and threshold at 4 kHz in children with frequent exposure to aircraft noise (20 flights overhead daily) as compared to matched controls [29]. However, the results of three other individual studies (a case-control study, a cross-sectional study, and a retrospective cohort study) did not show a significant association between aircraft noise and hearing loss in a pediatric population [30,31,32].

A systematic review on exposure to traffic noise and hearing loss was published more recently in 2012 [33]. It was shown that equivalent noise levels inside of the residences located near the highway ranged between 61–87 dBA, while the levels of noise in houses located away from the major traffic in the city ranged between 48–63 dBA. Accordingly, the audiometric testing showed hearing impairment defined as average threshold measured for low, middle and high frequencies greater than 25 dB HL in 81% of the exposed population and in 61% of the population living in dwellings located further away from major in the city. The noise dose (8 h %) of exposed subjects ranged between 54% and 103%, indicating an increased risk of hearing loss in some residents. The review includes only two field survey studies, carried out in 2005 by the same group of authors in India. The study populations were not defined, and sample sizes were not reported. In addition, the results of the study could be biased by confounding factors, such as potential population differences with respect to e.g., socio-economic status or working conditions in the subgroup living closer to major roads. Thus, this study does not allow the conclusion that the differences in the percentage of hearing loss were indeed caused by traffic noise exposure differences, or other causes.

### 1.3. Impulse Noise

A separate category of noise hazards is exposures to impulse noise, produced for example by guns or firecrackers. According to the Policy Interpretation Network on Children’s Health and Environment (PINCHE) report some toys can produce high impulse noise, e.g., toy cap guns, with peak levels ranging between 105 and 155 dBC in 25 cm distance [34]. Impulse noise is much more dangerous than steady-state noise; it can result in acute acoustic trauma and sudden irreversible hearing loss. Several international standards regulate such exposures. As for the toys, two international standards, the AS/NZS ISO 8124.1:2010 (and 2013) and the EN71 of the European Committee for Standardization (CEN) set limits for toys depending on their type [35,36,37]. The peak noise level for any toy (with the exception of percussion cap toys) should not exceed L_Cpeak_ = 115 dBC at a measuring distance of 50 cm. Consequently, the C-weighted peak level of “explosive” toys, such as cap guns that use powder, percussion caps, or other explosive methods, should not exceed the value of 125 dB (L_Cpeak_ ≤ 125 dB).

The available systematic reviews aimed at assessing the relationship between exposures to non-occupational noise in the general population and permanent hearing outcomes are very limited in number. The quality of evidence was low [33], the review was published more than five years ago [17], or was dedicated to a pediatric subpopulation [28], and the threshold levels of exposures increasing the risk for NIHL have not been established so far. Therefore, the main objective of this paper was to provide an overview of randomized and non-randomized studies and to determine whether the exposure-response relationship can be established between exposures to non-occupational noise (measured in sound pressure levels, SPLs, and expressed in L_Aeq8h_ values) and permanent hearing outcomes, such as permanent hearing loss and permanent tinnitus. The overarching goal was to provide actionable guidance for the prevention of NIHL in general population.

## 2. Materials and Methods

**Figure 1:**
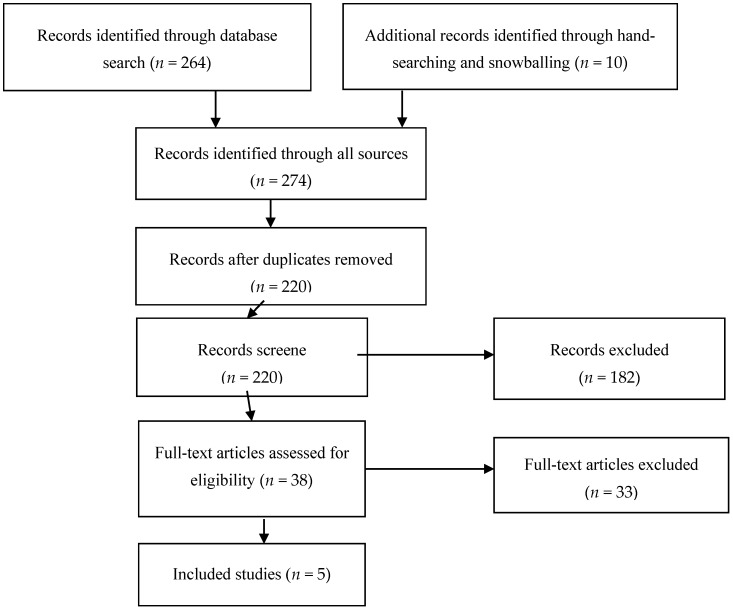
Flowchart of assessment of eligible studies.

The original papers were identified by a literature search of all of the accessible medical and other databases (PubMed, Web of Science, Scopus) using the terms “environmental noise, exposure levels, pubs, bars, concerts, sport events, personal listening devices (PLDs), toys, firecrackers, traffic noise, transport noise, combined exposures, noise-induced hearing loss (NIHL), hearing impairment, permanent hearing impairment, acoustic trauma, tinnitus, permanent tinnitus”. The conditions of the database search for personal listening devices were as follows: “personal [All Fields] AND (“auscultation” [MeSH Terms] OR “auscultation” [All Fields] OR “listening” [All Fields] OR “auditory perception” [MeSH Terms] OR (“auditory” [All Fields] AND “perception” [All Fields]) OR “auditory perception” [All Fields]) AND (“instrumentation” [Subheading] OR “instrumentation” [All Fields] OR “devices” [All Fields] OR “equipment and supplies” [MeSH Terms] OR (“equipment” [All Fields] AND “supplies” [All Fields]) OR “equipment and supplies” [All Fields]) AND (“hearing loss” [MeSH Terms] OR (“hearing” [All Fields] AND “loss” [All Fields]) OR “hearing loss” [All Fields]) AND exposure [All Fields] AND levels [All Fields] AND (“tinnitus” [MeSH Terms] OR “tinnitus” [All Fields])”. The papers identified in this way were analyzed in terms of inclusion criteria. The search was conducted until 30 June 2015, and it was not limited to a particular time span, except for the effects of PLDs. The latter was limited to the years 2008–June 30, 2015, since previous knowledge was summarized by SCENIHR descriptive systematic review published in 2008. Flowchart of assessment of eligible studies in given in Figure 1.

The inclusion criteria were as follows: the exposure to noise was expressed in individual decibel values (L_Aeq8h_), the studies included exposed and reference groups, the outcome was a permanent health effect, i.e., permanent hearing loss assessed with audiometry (or other quantifiable hearing test) or permanent tinnitus assessed with a questionnaire (Supplementary Materials, Table S1). Out of 38 full-text articles, 33 were excluded, mainly due to the lack of information on the noise level or the lack of audiometric examinations.

The search was performed according to the guidelines published in the report from “The evidence review meeting for the WHO Environmental Noise Guidelines for the European Region” held in Bern, Switzerland, from 29 September to 1 October 2014. According to this guideline, the risk of bias of the paper was assessed using the template for assessment of quality and risk of bias (Supplementary Materials, Table S2). The GRADE (grading of recommendations assessment, development and evaluation) approach was used to assess the overall quality of evidence (Supplementary Materials, Table S3) [38]. Taking into account factors decreasing quality of evidence (such as study limitations, inconsistency of results, indirectness of evidence, and imprecision) and factors increasing quality of evidence (such as large magnitude of effect, plausible confounding, which would reduce a demonstrated effect, presence of exposure-response gradient), the outcomes were graded as follow:

High quality—Further research is very unlikely to change our confidence in the estimate of effect;
Moderate quality—Further research is likely to have an important impact on our confidence in the estimate of effect and may change the estimate;
Low quality—Further research is very likely to have an important impact on our confidence in the estimate of effect and is likely to change the estimate; and,
Very low quality—Any estimate of effect is uncertain.

Extracted data included: (1) study design and the number of subjects; (2) age of subjects; (3) exposure assessment; (4) health outcome and the method of measurement; (5) the number (percentage) of subjects with hearing loss/tinnitus; (6) data analysis; (7) main results and conclusions; and (8) the risk of bias of the study.

The primary outcome measures were effect-size estimates, such as relative risk (RR) or odds ratio (OR) of permanent hearing loss and tinnitus related to exposure to environmental noise. In the case that the authors of a study provided the prevalence of hearing loss or tinnitus, but not the odds ratio (or relative risk), these values were additionally calculated by the authors of this review by logistic regression (non-linear estimation) with the use of STATISTICA (version 6, StatSoft, Inc.: Tulsa, OK, USA) software. Meta-analysis was not possible due to the methodological heterogeneity of included studies and the inadequacy of data.

## 3. Results

### 3.1. This Study Selection and Characteristics

After excluding duplicates, a total of 220 papers relevant by title to the subject of this study were identified. The inclusion statistics are listed by source of noise in Table 3.

Only five individual studies met the inclusion criteria and all of them were related to PLDs usage. The risk of bias of evaluated papers ranged from high to very high (Table 4). Two out of five papers refer to the relationship between listening to music through PLDs and permanent hearing loss. Another two refer to the relationship between the use of PLDs and tinnitus. One study addresses both permanent hearing loss and tinnitus. Two studies were performed by the same group of authors.

All five studies are cross-sectional and comprise in total of 1551 participants, including non-exposed controls [21,25,39,40,41]. Noise from PLD was estimated based on the direct measurement of equivalent sound pressure levels (in dB) in four studies [21,25,39,40], or based on converting volume-control setting levels of PLD into dB levels in one study [41]. In the case of direct measurement, the L_eq_ values were taken from the two 32-s measurements [39], from the 40-s measurement [25,40] or from 2-min measurement [21] of music sounds at typical and maximum level volume of the device. In all of the studies, individual L_Aeq8h_ value was calculated based on the estimated level of music and the number of hours a day listening to the music through the PLD declared by an individual in the questionnaire.

Exposure levels (L_eq_ values) had a mean of 72 dB [25], 81 dB [40], and 91 dB [21]. In two studies, this data was not provided [39,41]. Respectively, L_Aeq8h_ mean values were 62 dB [25], 76 dB [40], and 83 dB [21], or this data was not provided [39,41]. They were reported as individual L_Aeq8h_ [21,25,40], individual L_eq(32s)_, and L_EX,8h_ estimates [39], or individual sound levels in dBA for 56 h per week [41]. The median exposure level of the control group in the study by Sulaiman et al. (2014) [40] was not measured, since the control group consisted of subjects who never or rarely used PLD.

### 3.2. Risk of Bias within Studies

All of the studies were burdened with a high risk of bias when assessed using the template for assessment of quality and risk of bias (Supplementary Materials, Table S4). None of the studies included in this review provide information about blinding the health outcome and/or exposure assessment.

The other risks of bias within studies are as follows:

- Feder et al. (2013) [39]—Low participation rate (only 11% of out of 237 invited subjects);
- Sulaiman et al. (2013) [25]—The selection of participants was most likely not random and the response rate was not provided;
- Lévesque et al. (2010) [21]—No inclusion or exclusion criteria were specified. No confounding factors were included. A lack of randomization. The diagnostic criteria of tinnitus were not specified;
- Vogel et al. (2014) [41]—No direct measurement of noise levels; lack of randomization. It is not clear what proportion of subjects with “permanent hearing-related symptoms” experienced permanent tinnitus. No validated method of tinnitus assessment;
- Sulaiman et al. (2014) [40]—No information provided about blinding the health outcome and/or exposure assessment.

### 3.3. Results of Individual Studies

Results of the studies are summarized in Table 4. The odds ratios of permanent hearing loss and tinnitus are shown in Table 5.

#### 3.3.1. Permanent Hearing Loss

There is high risk of biased data indicating a positive correlation between individually measured L_Aeq8h_ values and hearing thresholds at 4 kHz, as well as low frequency pure-tone average at 0.5, 1 and 2 kHz (LFPTA). The individuals with reported use of 1–3 years and more than five years had higher hearing thresholds averaged over 4 and 8 kHz (high frequency pure-tone average, HEPTA) than the reference group using the PLDs for less than one year. This observation is based on one cross-sectional individual study performed in 237 Canadian teenagers [39]. The level range of music is not provided.

There is a high risk of bias data contradicting any significant association between subjects’ L_Aeq8h_ exposure levels and the incidence of permanent hearing loss assessed by standard pure-tone audiometry, or the occurrence of notched audiograms. However, individually measured L_Aeq8h_ values are positively correlated with hearing thresholds at two of the extended high frequencies (11.2 and 14 kHz), but not with the standard frequencies. This observation is based on one cross-sectional individual study performed in 177 Malaysian teenagers [25]. L_Aeq8h_ levels of music ranged from 43.0 dBA to 94.0 dBA (mean 61.6 ± 12.9 dBA).

There is high risk of biased data indicating a significant association between the PLDs usage (defined as using a PLD for at least 1 year, over 1h/day, and at over 50% of the maximum volume setting) and the incidence of hearing loss (defined as hearing thresholds ≥ 25 dB at one or more standard frequencies) and odds ratio of hearing loss (see Table 5). The conversion of volume-control levels into decibel levels (done according to Portnuff et al. (2011) [18]) showed that users listened to music at >70 dBA. Weak but significant correlation exists between users’ L_Aeq8h_ exposure levels and their PTA thresholds pooled from 3, 4, and 6 kHz. However, this association was found only in the right ear. Hearing thresholds of PLDs users at extended high frequencies are significantly higher than in non-users. Transient Evoked otoacoustic Emission (TEOAE) and Distortion Product Otoacoustic Emission DPOAE amplitudes in users are significantly reduced when compared with controls (non-PLDs users). This observation is based on one cross-sectional individual study performed in 35 young Malaysian adult PLDs users and an equal number of their age and sex matched controls, which never or rarely used PLDs [40]. L_Aeq8h_ levels of music ranged from 57.5 dBA to 89.7 dBA.

#### 3.3.2. Permanent Tinnitus

There is very high risk of biased data indicating a significant difference in the prevalence of tinnitus between groups using their PLDs at the L_Aeq8h_ ≤ 80 dBA and the L_Aeq8h_ > 80 dBA. However, there is no significant difference in the prevalence of tinnitus when the L_Aeq8h_ reference value is set at 85 dBA. When the prevalence values were recalculated into odds ratios by the authors of this review, no difference between differently exposed groups was found. The same was true when the limit was set at 80 dBA and at 85 dBA. This observation is based on one cross-sectional study performed in 124 Canadian teenagers [21]. L_Aeq8h_ levels of music ranged from 51.45 dBA to 110.34 dBA (mean 82.59 ± 11.89 dBA).

There is a high risk of biased data contradicting any significant difference in prevalence of tinnitus between groups listening to music through PLDs at the L_Aeq8h_ ≤ 75 dBA and the L_Aeq8h_ > 75 dBA. This observation is based on one cross-sectional study performed in 177 Malaysian teenagers using PLDs for at least six months prior the examination [25]. Similarly, when the prevalence values were recalculated into odds ratio by the authors of this report, no difference between groups was found. L_Aeq8h_ levels of music ranged from 43.0 dBA to 94.0 dBA.

Contrary to expectations, there is a high risk of biased data indicating that students not experiencing permanent hearing-related symptoms (possibly permanent tinnitus) listen to high-risk sound levels of music (equivalent to ≥90 dBA for 56 h per week) 2.5 times more often than the reference group (L_Aeq56h/week_ < 80 dBA). This observation is based on one cross-sectional study performed in 1228 Dutch teenagers and young adults [41]. LAq56h/week levels of music ranged from below 80 dBA to above 90 dBA.

### 3.4. Synthesis of Results

#### 3.4.1. Permanent Hearing Loss

None of the studies published up to date reported specific threshold analyses focused on stratifying the risk of permanent hearing loss due to PLDs use according to the levels of exposure to music.

The data of only one study of a high risk of bias with very low number of participants shows that the incidence and the risk of permanent hearing loss in regular PLDs users is significantly higher than in non-users (Table 5).

All three of the individual studies reporting on permanent hearing loss that are included in this review show a positive association between individual L_Aeq8h_ values and hearing thresholds at standard or extended audiometric frequencies. It is important to mention that two of these studies were published by the same group of authors.

Due to the methodological heterogeneity of the studies by Feder et al. (2013) [39] and Sulaiman et al. (2013, 2014) [25,40] pooled analysis was not possible.

#### 3.4.2. Permanent Tinnitus

The studies included in this report contradict any positive association between the equivalent levels of exposure to music listened to through the PLDs (averaged over 8 h a day or 56 h a week) and the incidence of permanent tinnitus, regardless of the cut-off level applied (75 dBA, 80 dBA, 85 dBA). Moreover, one of these studies indicates a negative association between listening to loud music through the PLDs at ≥90 dBA and permanent hearing-related symptoms, including permanent tinnitus. 

Pooled analysis was not performed due to an inadequacy of data. Levesque et al. (2010) [21] and Sulaiman et al. (2013) [25] did not provide criteria for the diagnosis of tinnitus, thus it is not known whether tinnitus was temporary or permanent; Vogel et al. (2014) [41] did not define how many of subjects with “permanent hearing-related symptoms” suffered permanent tinnitus.

#### 3.4.3. Risk of Bias across Studies

In all of the studies, the L_Aeq8h_ or L_Aeq56h/week_ levels of music were calculated based on a single sound pressure level measurement, which was performed in a quiet environment. Thus, the values calculated this way could be encumbered with error due to listener’s changing habits, using different types of devices and earphones, as well as changing the frequency of PLDs usage with high background noise. It has been shown that maximum sound pressure level of music listened through in-ear earphones differ by 14.4 dB depending on the style of music [24]. The level of music sound may increase from 6 to 10 dBA with background noise [23,42], and by 7–9 dB with using in-ear earphones when comparing with headphones [17,24].

According to the ISO 1999 model, the risk of hearing loss and the degree of hearing threshold shift depend on a combination of four major variables: age, sex, equivalent SPL, and years of exposure. In none of the studies included in this review, such comprehensive, combined analysis referring the measured hearing thresholds to those predicted by the ISO 1999 model was performed. The risk of permanent hearing loss was not analyzed separately for men and women.

Hearing loss in teenagers and young adults is the resultant value of combined exposures to different sources of noise including, but not limited to, PLDs. Therefore, other sources of music (discotheques, live concerts) represent the main bias risk and can additionally account to hearing thresholds shifts in PLD users. This issue was not directly addressed by the studies included in this review.

## 4. Discussion

### 4.1. Summary of Evidence

Among all of the sources of environmental noise, exposure to loud music seem to be the most frequent and possibly the most harmful, especially for young people. Up to date, the only data available on exposure-response relationship between exposure to loud music or other types of environmental sounds and permanent hearing outcomes is related to PLDs usage. These studies have been performed mainly in teenagers and young adults.

Noise-induced hearing loss develops very slowly over the years of exposure. Thus, the effects of overexposure to loud music could be difficult to single out in teenagers with a relatively short duration of PLDs usage. For example, according to the ISO model, a shift of hearing threshold greater or equal to 25 dB in speech frequencies should not take place in males with healthy ears, provided the exposure to noise does not exceed 15 years for 85 dBA level and six years for 90 dBA level. On the other hand, the dynamics of NIHL development is the greatest in the first years of exposure. For example, the magnitude of NIHL after 3 years is 40% of the NIHL after 45 years. Extended high frequency audiometry and otoacoustic emissions are believed to be more sensitive in the diagnostics of early stages of NIHL than standard audiometry.

The literature regarding recreational exposures to loud sounds, including listening to music through PLDs, is quite large. However, the design of a majority of the studies was to measure the levels of exposure and to predict the risk of hearing loss based on international standards, or to measure audiometric and tinnitus outcomes in the PLDs users versus non-users. The combination of both measures (exposure and hearing effect) was less frequent and based mainly on self-reported questionnaire information or in relation to temporary hearing outcomes. Thus, the data on the exposure-response relationship between the exposure to music listened to through PLDs and permanent hearing loss measured by a quantifiable hearing test are very scarce. So far, only three studies of high risk of bias were published by two groups of researchers. The studies were performed in Malaysian and Canadian populations. However very limited, they consistently indicate that exposures to loud music listened to through PLDs may result in hearing threshold shifts, provided the level of music is high and the duration of exposure is long (more than five years). The results of these studies show that if the exposure to music is relatively short (mean 3.2 years), the typical signs of NIHL are not detected in the standard audiometric frequencies (0.25–8 kHz), but can be visible at extended high frequencies [25]. As anticipated, the early stages of NIHL can also be recognized by decreasing the signals of otoacoustic emissions [40]. Using PLDs for a longer time was shown to be associated with an increased incidence of permanent hearing loss and the worsening of hearing thresholds at standard test frequencies related to the exposure level [39,40]. This was distinctly observed at high standard audiometric frequencies, including the frequency of 4 kHz, which is most sensitive for acoustic trauma.

In none of the studies included in this review was a limit of exposure to music established. Some previous literature data indicate that the instance of audiometric notch was considerably higher in young people studying popular music than reported from studies of the general population [43]. On the other hand, professional musicians who do not use hearing protectors seem to develop less profound hearing loss than predicted based on the occupational noise standards [44]. Therefore, it can be speculated that since music is a “desired” sound, it may be less harmful than occupational noise.

NIHL is very often accompanied by tinnitus. None of the studies included in this review support the hypothesis that the incidence of permanent tinnitus is positively associated with exposures to loud music. However, in this review the risk of bias of all three studies on tinnitus was high or very high [21,25,41]. Thus, this conclusion should be drawn with caution. The findings indicating that the incidence of permanent hearing-related symptoms is negatively correlated with high risk exposure to music listened to through PLDs can be coincidental or can be the effect of ear “conditioning”. In either case, this outcome should be verified.

The data of this review suggests that the OR of permanent hearing loss might be significantly (about four times) higher in regular PLDs users versus non-users. The users are defined as these listening to the music through PLDs for at least 1 h per day, at more than 50% of the maximum volume setting, for at least one year [40]. Similar times of exposure expressed in hours per week were shown by other studies [45,46]. Only one study provides more a conservative value of 4 h per week [47]. The literature data shows also that the critical limit for the safety of exposure to loud sounds of music is L_eq_, 8 h of 80 dBA [46], and the hearing effects of using PLDs could be most clearly seen after five years of exposure [48,49]. Thus, for public health activities, the conclusive statement of the SCENIHR report on the safety of PLDs seems to be still valid. Attention should be paid if an individual is exposed to high level of music (over 50% of PLD volume setting) for more than 1 h a day [17].

To our knowledge, currently there are no papers meeting the inclusion criteria applied in this work, which would indicate a potential relationship between other environmental noise sources and hearing loss.

### 4.2. Limitations

The literature on the exposure-response relationship between the exposures to environmental noise and permanent hearing outcomes such as hearing loss/tinnitus is limited.

As for the PLDs usage, the results of the studies included in this review should be interpreted with caution due to the high risk of bias of the studies, as well as the possible limitations of using the occupational noise standard to predict hearing consequences of relatively short exposures to leisure music.

Large-scale case-control studies and longitudinal cohort studies are needed to address the objective of this review in the future. It should be stressed that such studies might be difficult to perform, because the assessment of the average long-term noise exposure appears to be largely impossible due to varying sound levels at different venues and changing individual listening habits over time. Sound levels in different situations and the daily usage of devices may also change, as well as monthly visits of entertainment places and venues. Another limitation is the long time of exposure required for measurable audiometric hearing loss.

As it has been shown that temporary threshold shift after exposure to noise might be reasonably predictive of future permanent threshold shift [50], it would be worth to proceed with the studies on TTS in the future. Up-to-date results show that exposure-effect relationship for TTS and tinnitus is feasible to be established [51].

An alternative approach to advance the studies on the relationship between exposure to music listened to through PLDs and the risk of permanent hearing loss and tinnitus would be to look at age-related hearing loss progression depending on early age exposures to loud music.

## 5. Conclusions

- While using very strict inclusion criteria, there is low quality GRADE evidence that prolonged listening to loud music through PLDs increases the risk of hearing loss and results in worsening standard frequency audiometric thresholds.
- Specific threshold analyses focused on risk stratification of permanent hearing loss according to clearly defined levels of exposure to music through the PLDs are missing. This is due to many restrictions of conducting such research that are related to the long latency time from exposure to effect, the problems to correctly estimate the exposure, and the lack of sensitive measures to detect early signs of hearing loss.
- Available, yet very limited, data contradict any positive association between prolonged listening to loud music through PLDs and the risk of permanent tinnitus. Future studies are needed to provide actionable guidance for PLDs users.
- No studies fulfilling the inclusion criteria related to other isolated or combined exposures to environmental noise were identified.
- Since measurable audiometric hearing loss requires long exposure periods and individual dosimetry is preferable to overcome the methodological and practical drawbacks in exposure assessment, such cohort studies would be very challenging.

## Figures and Tables

**Table 1:** The examples of equivalent time-intensity levels referred to action levels according to Directive 2003/10/EC [7].

Action Level	L_EX,8h_	Equivalent Levels for Time Indicated (Trade-Off 3 dB)
First Action level (minimum) provide protection	80 dB(A)	83 dBA-4 h *; 86 dBA-2 h; 89 dBA-1 h;
		92 dBA-30 min **; 95 dBA-15 min; 98 dBA-8 min;
		101 dBA-4 min; 104 dBA-2 min; 107 dBA-1 min
Second Action level mandatory protection	85 dB(A)	88 dBA-4 h; 91 dBA-2 h; 94 dBA-1 h;
		97 dBA-30 min; 100 dBA-15 min; 105 dBA-5 min;
		111 dBA-1 min
Maximum Exposure limit value	87 dB(A)	90 dBA-4 h; 93 dBA-2 h; 96 dBA-1 h;
		99 dBA-30 min; 102 dBA-15 min; 107 dBA-5 min;
		113 dBA-1 min

* hours; ** minutes.

**Table 2:** Risk of permanent hearing loss due to the use of Personal Listening Devices (studies published after 2007).

No	Author, Year	Population	Exposure	References Group	Confounding	Outcome
1.	Keith et al. (2011) [22]	248 Canadian individuals aged 10–18, 110 males and 138 females, 29 subjects excluded, random school and student selection.	Individual L_EX_ was calculated according to ISO 1999, open-ended question about the hours per week listening to music, weekly allowable listening duration time was divided by seven to derive an estimate of average daily listening duration, measurement made in classrooms, for 32 s listening of music at the typical and “worst-case” volume levels, background noise between 40 and 52 dBA.	ISO 1990	All uncertainty estimates were based on the ISO/IEC guide (ISO/IEC, 1995). Low participation rate of 11%.	3.2% of subjects were estimated to exceed the level limit of 85 dBA L_EX_ of the typical volume settings. 77.5% of listeners were exposed to the level for which there is no known risk of permanent noise induced hearing loss, i.e., ≤75 dBA L_EX_.
2.	Portnuff et al. (2013) [19]	52 US individuals, aged 18–29, hearing threshold no worse than 20 dB HL, at least 10 h use of PLD a week, 24 from this group were chosen at random for dosimetry.	An earphone data logging system connected with the dosimeter was developed in order to record the real-world use of PLD, dose calculation: self-reported chosen listening level (CLL) (Dose usual), the CLL by volume control increments (Dose vol) and the measured dose from the logg in system (Dose measured).	NIOSH, OSHA	Bias due to participants selection (listening for at least 10 h a week).	Weekly damage risk criteria were exceeded in 16.7% subjects and 8.3% of subjects according to NIOSH and OSHA standards, respectively.
3.	Portnuff et al. (2011) [18]	29 US individuals (12 males and 17 females), aged 13–17, using MP3 players at least two hours per week, normal hearing thresholds (no worse than 15 dB HL).	Individual noise doses calculated based on self-reported listening time and self-reported volume control setting (based on translation of volume control settings to diffuse-field equivalent output levels).	EPCEU, NIOSH and OSHA criteria	No direct SPL measurement.	Doses of noise higher than damage risk criteria in 0%—OSHA, in 6.9%—NIOSH, 13.8%—EPCEU of subjects.
4.	Lee et al. (2014) [27]	1928 Singapore university freshman, aged 16–21 years, different races, 129 students excluded, 95.8% regular PLD users.	Based on volume setting and questionnaire. Pre-calibrated MP3 players, so the equivalent A-weighted SPL of the different volume settings was known	TWA 8 h > 85 dBA	No direct SPL measurement	16.4% students were exposed at TWA 8 h > 85 dBA. Differences between races—Chinese less exposed.
5.	Muchnik et al. (2012) [23]	74 Israeli individuals (26 males and 48 females), aged 14–16 year, regular PLDs users, no history of hearing problems and middle ear pathologies.	Preferred listening levels of six types of PLDs. Volume control setting transformed to SPLs which were in turn converted to equivalent diffuse field A-weigthed levels, ambient noise 61–70 dBA. L_Aeq_ 85 dBA were calculated.	NIOSH	No direct SPL measurement. Measurement done in the presence of background noise.	Mean preferred listening level 89 (SD—9) dBA. 26% of the participants in the noisy condition were found to be at risk according to occupational damage risk NIOSH criteria (NIOSH 1998).
6.	Vogel et al. (2011) [14]	1687 Dutch students from 15 Dutch secondary schools invited, response rate 89.9% (1512 subjecrts, 89.9% of tjose PLD users)	Average weekly exposure time to MP3 players was estimated by referring the volume of the device to dB(A) value and multiplying days per week and hours per day to calculate (weekly) Permissible Exposure Limits (PELweek = music level of 89 dBA listen for 56 h a week).	89 dBA for ≥1 h per day. Reffered to the safe level of 80 dBA (like in SCENIHR report) converted to L_EX,w_ of 56 h.	No direct SPL measurement.	28.6% of risk 89 dBA ≥ 1 h.

**Table 3:** Statistics of included and excluded studies.

	Source	Leisure Steady-State Noise/Music				Impulse Noise	Aircraft/Traffic Noise and Other Exposures	Total
Number of Studies		PLD	Bars	Concerts	Sport Events	Toys, Firearms & Firecrackers		
Included studies		5	0	0	0	0	0	5
Excluded studies		116	15	68	6	37	12	215 *
Total		121	15	68	6	37	12	220 *

* Numbers of total studies are not equal to the sum of these studies listed by the source of noise due to several sources of noise reported by some studies.

**Table 4:** Results of individual studies fulfilling the inclusion criteria.

No	Author and Year	Study Design and the Number of Subjects	Age of Subjects (Year)	Exposure Assessment	Health Outcome and the Method of Measurement	The Number (Percent) of Subjects with Hearing Loss/Tinnitus	Data Analysis	Main Results and Conclusions	Risk of Bias
**1.**	Feder et al. (2013) [39]	Cross-sectional (*n* = 237)	10–17	Individual L_eq(32 s)_ and L_EX,8h_ estimates	Permanent hearing loss; PTA	Data not reported	Multivariate models for audiometric frequencies (estimate (SE), *p*-value from *t*-test based on multivariate model).	Positive correlation between Lex(8 h) and hearing threshold at 4 kHz and LFPTA (low frequency pure-tone average for frequencies 0.5, 1 and 2 kHz) Subjects reporting using their player for >5years and those reporting using their player for 1 to <3 years both had higher HT than those using their player for <1 year	High, due to low subject participation rate (only 11%) and no information provided about blinding the health outcome and/or exposure assessment
**2.**	Sulaiman et al. (2013) [25]	Cross-sectional (*n* = 177)	13–16	Individual L_Aeq8h_	1. Permanent hearing loss; standard PTA and extended high frequencies PTA 2. Tinnitus (possibly permanent)	Hearing loss found in 13 (7.3%) of subjects in entire population. Tinnitus reported by 30 (16.9%) subjects exposed to music at L_Aeq8h_ ≤ 75 dBA and by 7 (4.0%) of those exposed to music at L_Aeq8h_ >75 dBA.	Pearson correlation test, Chi-squared test	No significant association between subjects’ L_Aeq8h_ exposure levels and the incidence of hearing loss (defined as HT ≥ 25 dB at one or more standard frequencies), or the occurrence of notched audiograms. Weak significant correlation between individual L_Aeq8h_ and HT at 11.2 and 14 kHz. No relationship between individual L_Aeq8h_ and tinnitus.	High, the response rate was not provided, neither the information about blinding the health outcome and/or exposure assessment.
**3.**	Sulaiman et al. (2014) [40]	Cross-sectional (35 exposed subjects and equal number of age and sex matched not exposed subjects)	18–30	Individual L_Aeq8h_	Permanent hearing loss; standard PTA and extended high frequencies PTA, otoacoustic emissions	Hearing loss (defined as HT ≥ 25 dB at one or more standard frequencies) in 12 (34.3%) of users and 4 (11.4%) of control subjects.	Descriptive analysis, unpaired Student *t* test, Pearson correlation test, Fisher’s exact test.	Significant association between the incidence of hearing loss and PLDs usage. Weak but significant correlation between users’ L_Aeq8h_ exposure levels and their PTA thresholds pooled from 3, 4 and 6 kHz in the right ear only. Hearing thresholds of PLDs users at extended high frequencies significantly higher than in non-users. Otoacoustic emissions amplitudes in users significantly reduced compared with controls.	High, no information provided about blinding the health outcome and/or exposure assessment.
**4.**	Lévesque et al. (2010) [21]	Cross-sectional (*n* = 124)	14–17	Individual L_Aeq8h_	Tinnitus (possibly permanent); questionnaire	Tinnitus reported by 2 (4.1%) of subjects exposed to music at L_Aeq8h_ ≤ 80 dBA and by 12 (16.0%) of those exposed to music at L_Aeq8h_ > 80 dBA. Tinnitus reported by 5 (6.9%) of subjects exposed to music at L_Aeq8h_ ≤ 85 dBA and by 9 (17.3%) of those exposed to music at L_Aeq8h_ > 85 dBA.	Binary outcome, chi2 test	Significant difference in prevalence of tinnitus between groups when the L_Aeq8h_ reference value set at 80 dBA. No significant difference in prevalence of tinnitus when the L_Aeq8h_ reference value set at 85 dBA.	Very high, due to the lack of specification of inclusion/exclusion criteria, participation rate 63.3%, diagnostic criteria of tinnitus not specified, no confounding factors included, no information provided about blinding the health outcome and/or exposure assessment.
**5.**	Vogel et al. (2014) [41]	Cross-sectional (*n* = 943)	16–25	Individual sound levels in dBA for 56 h per week, estimated based on PLD volume setting (Permissible Exposure Limits (PELweek = music level of 89 dBA listen for 56 h a week)	Permanent hearing-related symptoms; questionnaire (not clear what proportion of subjects with “permanent hearing-related symptoms” experienced permanent tinnitus)	Permanent hearing-related symptoms reported by 101 (10.7%) of subjects not at risk (<80 dBA), 93 (9.9%) of subjects at low risk (80–85 dBA), 97 (10.3%) of subjects at moderate risk (85–90 dBA) and 41 (4.4%) of subjects at high risk (≥90 dBA).	Multiple logistic regression; ORs: 0.86 (0.49–1.46), 0.93 (0.50–1.75) 0.39 (0.18–0.86) for low risk (80–85 dBA), moderate risk (85–90 dBA) and high risk (≥90 dBA) group of listeners.	Students not experiencing permanent hearing-related symptoms listen > 2.5 times more often to high-risk sound levels (equivalent to ≥ 90 dBA for 56 h per week).	High, because of the lack of direct measurement of sound pressure levels, health outcome assessment leading to information bias.

**Table 5:** Odds ratios (95% confidence intervals, CI) of tinnitus/permanent hearing-related symptoms depending on reference value (L_Aeq8h_) and permanent hearing loss in users vs. non-users.

No	Author and Year	Health Outcome	Reference Values (L_Aeq8h_)	OR	(95% CI)	Number of Participants	Risk of Bias
1.	Sulaiman et al. (2013) [25]	Tinnitus	≤75 dBA	1.00		177	High
			>75 dBA	1.13 ^1^	(0.44—2.89) ^1^		
2.	Levesque et al. (2010) [21]	Tinnitus	≤80 dBA	1.00		124	Very high
			>80 dBA	4.48 ^1^	(0.94–21.29) ^1^		
			≤85 dBA	1.00			
			>85 dBA	2.80 ^1^	(0.87–9.04) ^1^		
3.	Vogel et al. (2014) [41]	Permanent hearing related symptoms (tinnitus)	<80 dBA	1.00		943	High
			80–85 dBA	0.86	(0.49–1.49)		
			85–90 dBA	0.93	(0.50–1.75)		
			≥90 dBA	0.39	(0.18–0.86)		
4.	Sulaiman et al. (2014) [40]	Permanent hearing loss *	non users	1.00		70	High
			Users **	4.04 ^1^	(1.13–14.49) ^1^		

^1^ Values calculated by the authors of this paper based on originally provided data on prevalence of tinnitus or permanent hearing loss. * HT ≥ 25 dB at one or more standard frequencies. ** Users of PLDs for at least 1 year, 1 h/day and at >50% of the maximum volume setting. HT—hearing thresholds.

## Supplementary Materials

The following are available online at www.mdpi.com/1660-4601/14/10/1139/s1, Table S1: Individual studies had to meet the following criteria in order to be included in the evidence review. If the criteria were adjusted, a justification is given detailing the reasons, Table S2: The template for assessment of quality and risk of bias, Table S3: GRADE for quality of evidence from personal listening devices associated with hearing impairment and tinnitus, Table S4: The template for assessment of quality and risk of bias of individual studies, Table S5: The list of included and excluded studies.

## References

[1] World Health Organization (WHO) Global Burden of Hearing Loss in the Year 2000. www.who.int/healthinfo/statistics/bod_hearingloss.pdf.

[2] Henderson D., Bielehed E.C., Harris K.C., Hu B.H. (2006). The role of oxidative stress in noise-induced hearing loss. Ear Hear..

[3] Puel J.L., Ruel J., Guitton M., Pujol R. (2002). The inner hair cell afferent/efferent synapses revisited: A basis for new therapeutic strategies. Adv. Otorhinolaryngol..

[4] Pilati N., Ison M.J., Barker M., Mulheran M., Large C.H., Forsythe I.D., Matthias J., Hamann M. (2012). Mechanisms contributing to central excitability changes during hearing loss. Proc. Natl. Acad. Sci. USA.

[5] Lin H.W., Furman A.C., Kujawa S.G., Liberman M.C. (2011). Primary neural degeneration in the guinea pig cochlea after reversible noise-induced threshold shift. J. Assoc. Res. Otolaryngol..

[6] Plack C.J., Barker D., Prendergast G. (2014). Perceptual consequences of “hidden” hearing loss. Trends Hear..

[7] European Commission (2013). Directive 2003/10/EC of the European Parliament and of the Council of 6 February 2003 on the Minimum Health and Safety Requirements Regarding the Exposure of Workers to the Risks Arising from Physical Agents (Noise).

[8] International Standard Organization (ISO) (1990). ISO 1999:1990: Acoustics—Determination of Occupational Noise Exposure and Estimation of Noise-Induced Hearing Impairment.

[9] Beach E., Williams W., Gilliver M. (2013). Estimating young Australian adults’ risk of hearing damage from selected leisure activities. Ear Hear..

[10] Johnson O., Andrew B., Walker D., Morgan S., Aldren A. (2014). British university students’ attitudes towards noise-induced hearing loss caused by nightclub attendance. J. Laryngol. Otol..

[11] Lewis R.C., Gershon R.R., Neitzel R.L. (2013). Estimation of permanent noise-induced hearing loss in an urban setting. Environ. Sci. Technol..

[12] Berg A.L., Serpanos Y.C. (2011). High frequency hearing sensitivity in adolescent females of a lower socioeconomic status over a period of 24 years (1985–2008). J. Adolesc. Health.

[13] Cone B.K., Wake M., Tobin S., Poulakis Z., Rickards F.W. (2010). Slight-mild sensorineural hearing loss in children: Audiometric, clinical, and risk factor profiles. Ear Hear..

[14] Vogel I., Brug J., Van der Ploeg C.P., Raat H. (2011). Adolescents risky MP3-player listening and its psychosocial correlates. Health Educ. Res..

[15] Pellegrino E., Lorini C., Allodi G., Buonamici C., Garofalo G., Bonaccorsi G. (2013). Music-listening habits with MP3 player in a group of adolescents: A descriptive survey. Ann. Ig..

[16] World Helath Organization (WHO) 2015 Hearing Loss due to Recreational Exposure to Loud Sound. A Review. http://apps.who.int/iris/bitstream/10665/154589/1/9789241508513_eng.pdf?ua=1.

[17] European Commission—Scientific Committee on Emerging and Newly Identified Health Risks (2008). Potential Health Risks of Exposure to Noise from Personal Music Players and Mobile Phones Including a Music Playing Function. http://ec.europa.eu/health/ph_risk/committees/04_scenihr/docs/scenihr_o_018.pdf.

[18] Portnuff C.D., Fligor B.J., Arehart K.H. (2011). Teenage use of portable listening devices: A hazard to hearing?. J. Am. Acad. Audiol..

[19] Portnuff C.D., Fligor B.J., Arehart K.H. (2013). Self-report and long-term field measures of MP3 player use: How accurate is self-report?. Int. J. Audiol..

[20] Williams W. (2009). Trends in listening to personal stereos. Int. J. Audiol..

[21] Lévesque B., Fiset R., Isabelle L., Gauvin D., Baril J., Larocque R., Gingras S., Leroux T., Picard M., Girard S.A. (2010). Exposure of high school students to noise from personal music players in Québec City, Canada. Int. J. Child. Adolesc. Health.

[22] Keith S.E., Michaud D.S., Feder K., Haider I., Marro L., Thompson E., Marcoux A.M. (2011). MP3 player listening sound pressure levels among 10 to 17 year old students. J. Acoust. Soc. Am..

[23] Muchnik C., Amir N., Shabtai E., Kaplan-Neeman R. (2012). Preferred listening levels of personal listening devices in young teenagers: Self reports and physical measurements. Int. J. Audiol..

[24] Breinbauer H.A., Anabalón B., Gutierrez D., Cárcamo R., Olivares C., Caro J. (2012). Output capabilities of personal music players and assessment of preferred listening levels of test subjects: Outlining recommendations for preventing music-induced hearing loss. Laryngoscope.

[25] Sulaiman A.H., Seluakumaran K., Husain R. (2013). Hearing risk associated with the usage of personal listening devices among urban high school students in Malaysia. Public Health.

[26] National Institute for Occupational Safety and Health (NIOSH) (1998). Criteria for Recommended Standard—Occupational Noise Exposure: Revised Criteria.

[27] Lee G.J., Lim M.Y., Kuan A.Y., Teo J.H., Tan H.G., Low W.K. (2014). The music listening preferences and habits of youths in Singapore and its relation to leisure noise-induced hearing loss. Singap. Med. J..

[28] Vasconcellos A.P., Kyle M.E., Gilani S., Shin J.J. (2014). Personally modifiable risk factors associated with pediatric hearing loss: A systematic review. Otolaryng. Head Neck.

[29] Chen T., Chen S. (1993). Effects of aircraft noise on hearing and auditory pathway function of school-age children. Int. Arch. Occup. Environ. Health.

[30] Green K.B., Pasternack B.S., Shore R.E. (1982). Effects of aircraft noise on hearing ability of school-age children. Arch. Environ. Health.

[31] Andrus W.S., Kerrigan M.E., Bird K.T. (1975). Hearing in para-airport children. Aviat. Space Environ. Med..

[32] Fisch L. (1981). Aircraft noise and hearing impairment in children. Br. J. Audiol..

[33] Banerjee D. (2012). Research on road traffic noise and human health in India: Review of literature from 1991 to current. Noise Health.

[34] European Commission, Community Research and Development Information Service (2005). The Policy Interpretation Network on Children’s Health and Environment (PINCHE) Project.

[35] AS/NZS ISO 8124.1 (2010). AS/NZS ISO 8124.1:2010: Safety of Toys, Part 1: Safety Aspects Related to Mechanical and Physical Properties (ISO 8124–1:2009).

[36] AS/NZS ISO 8124.1 (2013). AS/NZS ISO 8124.1:2013: Safety of Toys, Part 1: Safety Aspects Related to Mechanical and Physical Properties (ISO 8124–1:2012).

[37] EN 71-1:2011+A2:2013 (2013). Safety of Toys—Part 1: Mechanical and Physical Properties.

[38] Guyatt G.H., Oxman A.D., Vist G.E., Kunz R., Falck-Ytter Y., Alonso-Coello P., Schünemann H.J. (2008). GRADE Working Group. GRADE: An emerging consensus on rating quality of evidence and strength of recommendations. BMJ.

[39] Feder K., Marro L., Keith S.E., Michaud D.S. (2013). Audiometric thresholds and portable digital audio player user listening habits. Int. J. Audiol..

[40] Sulaiman A.H., Husain R., Seluakumaran K. (2014). Evaluation of early hearing damage in personal listening device users using extended high-frequency audiometry and otoacoustic emissions. Eur. Arch. Otorhinolaryngol..

[41] Vogel I., van de Looij-Jansen P.M., Mieloo C.L., Burdorf A., de Waart F. (2014). Risky music listening, permanent tinnitus and depression, anxiety, thoughts about suicide and adverse general health. PLoS ONE.

[42] Hodgetts W.E., Riecker J.M., Szarko R.A. (2007). The effects of listening environment and earphone style on preferred listening levels of normal hearing adults using an MP3 player. Ear Hear..

[43] Barlow C. (2011). Evidence of noise-induced hearing loss in young people studying popular music. Med. Probl. Perform. Art..

[44] Pawlaczyk-Luszczynska M., Dudarewicz A., Zaborowski K., Zamojska M., Sliwinska-Kowalska M. (2013). Noise induced hearing loss: Research in central, eastern and south-eastern Europe and newly independent states. Noise Health.

[45] Meyer-Bisch C. (1996). Epidemiological evaluation of hearing damage related to strongly amplified music (personal cassette players, discotheques, rock concerts) high-definition audiometric survey on 1364 subjects. Audiology.

[46] Marron K.H., Sproat B., Ross D., Wagner S., Alessio H. (2014). Music listening behavior, health, hearing and otoacoustic emission levels. Int. J. Environ. Res. Public Health.

[47] Weichbold V., Holzer A., Newesely G., Stephan K. (2012). Results from high-frequency hearing screening in 14- to 15-year old adolescents and their relation to self-reported exposure to loud music. Int. J. Audiol..

[48] Peng J.H., Tao Z.Z., Huang Z.W. (2007). Risk of Damage to Hearing from Personal Listening Devices in Young Adults. J. Otolaryngol..

[49] Kim M.G., Hong S.M., Shim H.J., Kim Y.D., Cha C.I., Yeo S.G. (2009). Hearing threshold of Korean adolescents associated with the use of personal music players. Yonsei Med. J..

[50] Moshammer H., Kundi M., Wallner P., Herbst A., Feuerstein A., Hutter H.P. (2015). Early prognosis of noise-induced hearing loss. Occup. Environ. Med..

[51] Vogel I., Verschuure H., van der Ploeg C.P., Brug J., Raat H. (2010). Estimating adolescent risk for hearing loss based on data from a large school-based survey. Am. J. Public Health.

