# Supplementary material for: WHO Environmental Noise Guidelines for the European Region: A Systematic Review on Environmental Noise and Permanent Hearing Loss and Tinnitus
Source: Int J Environ Res Public Health. 2017 Sep 27;14(10):1139. doi: 10.3390/ijerph14101139 (PMC5664640; doi:10.3390/ijerph14101139)
Supplement: Supplementary file 1 [file ijerph-14-01139-s001.pdf]

International Journal of
Environmental Research and Public Health

MDPI

WHO Environmental Noise Guidelines for the European Region: A Systematic Review on Environmental Noise and Permanent Hearing Loss and Tinnitus

Mariola Śliwińska-Kowalska [1,*] and Kamil Zaborowski [2]

1. Inclusion and Exclusion Criteria for Individual Studies

Table S1. Individual studies had to meet the following criteria in order to be included in the evidence review. If the criteria were adjusted, a justification is given detailing the reasons.

	Inclusion Criteria	Exclusion Criteria
**Population**: general population in settings (hospitals, residences, public venues, educational facilities)	- Studies including members of the general population<br>- Studies including specific segments of the population particularly at risk, such as children or vulnerable groups<br>- Studies including participants exposed to noise in occupational settings if relevant with combined exposure to environmental noise only	- Does not meet inclusion criteria<br>- Studies including participants exposed to noise in occupational settings not relevant with combined exposure to environmental noise
**Exposure**: exposure to high levels of environmental noise from various noise sources	- Noise exposure levels either measured or calculated and expressed in decibel values. They should aim to be representative of the individual exposure of the study participants.	- Does not meet inclusion criteria<br>- Studies using hearing loss or defective hearing as a proxy for (previous) noise exposure<br>- Surveys assessing noise exposure on the basis of subjective ratings, as given by the subjects in a questionnaire
**Comparator**: no noise exposure or lower levels of noise exposure	- Should have comparator group (corresponding to no exposure or lower level exposure)	- Does not meet inclusion criteria
**Confounding**: adjusted for confounding	- No inclusion criteria applied; however, for every study, we will assess which possible confounders have been taken into account	- No exclusion criteria applied
**Outcome**: assessment of outcome	- Permanent threshold shift measured with pure-tone audiometry and permanent tinnitus assessed with the questionnaire;<br>- Relative risks (RR) or odds ratios (OR) as primary outcome measures	- Does not meet inclusion criteria

Table S2. The template for assessment of quality and risk of bias.

**Risk of Bias Check List for Studies on Noise and Health Outcomes**	**Study Name:**	**Assessor Name:**	**Date Assessed:**
Study Design	Cohort<br>Case-control<br>Cross-sectional		
Domain	Description of criteria for judgment	Quote from article on which the judgment is based	Judgment of risk of bias
Noise Exposure assessment leading to information bias			
	The noise level (in decibel) is expressed in Lden and Lnight or its components (Lday, Levening, Lnight and the duration in hours of Lnight) AND:<br>For long term average noise level:<br>A. is based on a noise map using as input the actual traffic volume, composition and speed per 24 h per road/railway /airport, or the type and sound power of an industrial installation and the size in terms of either production volume or persons employed<br>OR<br>B. is based on measurements for a minimum of 1 week by qualified staff, and adjusted for data under point A. as well as meteorological conditions when necessary<br>OR<br>C. is based on a noise map reported in a separate publication but which fulfils conditions A or B<br>For short-term noise level:<br>D. is based on measurements for a sufficient time by qualified staff		Low
	The noise level is not expressed in decibels OR is not expressed in Lden and Lnight or its components<br>OR:<br>For long term average noise level:<br>A. is based on a map that does not use as input the actual traffic volume, composition and speed per 24 h per road/railway/airport, or the type and sound power of an industrial installation and the size in terms of either production volume or persons employed<br>OR<br>B. is based on measurements of less than 1 week OR not adjusted for data under point A. or meteorological conditions when necessary OR by unqualified staff<br>OR<br>C. is based on a noise map reported in a separate publication but which does not fulfils conditions A or B<br>For short-term noise level:<br>D. is based on measurements for an insufficient time OR by unqualified staff		High
	If not enough information reported, to judge the above		Unclear
Bias due to confounding			

At least the following confounders should be incorporated for a valid assessment for the relation between noise and XXX outcome: 1….2….3….4….	All important confounders taken into account either through matching, restriction or in the analysis	Low
	Only 1 or no confounder taken into account OR Subjects in exposed and unexposed groups differ for one or more important confounders and there is no adjustment in the analysis	High
	Less then all to >1 important confounders taken into account OR Insufficient information to decide on one of the above	Unclear
Bias due to selection of Participants		
	Participants randomly sampled from a known population AND response rate higher than 60% AND attrition rate less than 20% in follow up studies	Low
	No random sampling OR response rate less than 60%	High
	No information to judge the above	Unclear
Health outcome assessment leading to information bias I		
	The health outcome of interest is objectively measured OR taken from medical records OR taken from questionnaire or interview using a known scale or validated assessment method	Low
	The health outcome of interest is self-reported and not assessed using a known scale or validated assessment method	High
	Not sufficient information reported to assess the above	Unclear
Health outcome assessment leading to information bias II		
	The health outcome of interest is assessed blind for exposure information in cohort and cross-sectional studies or exposure is assessed blind for being a case in case-control studies	Low
	The health outcome and/or exposure assessment is not blinded	High
	Not sufficient information reported to assess the above	Unclear
	Total Risk of Bias in Study	
	At least 4/5 at low risk of bias, including for domains 1, 2 and 3	Low
	Any other	High

Table S3. GRADE for quality of evidence from personal listening devices associated with hearing impairment and tinnitus.

**Hearing Impairment**			
**Domains**	**Criterion**	**Assessment**	**Downgrading**
Start Level	Longitudinal = high; others = low	All cross-sectional studies	Low quality
1. Study Limitations	Majority of studies low quality	All with high risk of bias	Downgrade one level
2. Inconsistency	Conflicting results; high I^2	Generally consistent results on association between environmental noise and permanent hearing loss	No reason for downgrading
3. Directness	Direct comparison; same PECO*	Yes, same PECO*	No reason for downgrading
4. Precision	Confidence interval contains 25% harm or benefit	All CI wider than 25%	Downgrade one level
5. Publication Bias	Funnel plot indicates	Not able to assess; too few studies	No downgrade
Overall judgment			Very low quality
6. Exposure-response	Significant trend	Not observed	No upgrading
7. Magnitude of effect	RR > 2	Not observed	No upgrading
8. Confounding adjusted	Effect in spite of confounding working towards the nil	Not observed	No upgrading
Overall Judgement			Very low quality

**Tinnitus**			
**Domains**	**Criterion**	**Assessment**	**Downgrading**
Start Level	Longitudinal = high; others = low	All cross-sectional	Low quality
1. Study Limitations	Majority of studies low quality	All studies with high risk of bias	Downgrade one level
2. Inconsistency	Conflicting results; high I^2	Contradictory results on association between environmental noise and permanent tinnitus	Downgrade one level
3. Directness	Direct comparison; same PECO*	Yes, same PECO*	No reason for downgrading
4. Precision	Confidence interval contains 25% harm or benefit	All CI wider than 25%	Downgrade one level
5. Publication Bias	Funnel plot indicates	Not able to assess	No reason for downgrading
Overall judgment			Very low quality
6. Exposure-response	Significant trend	Not possible to assess	No reason for upgrading
7. Magnitude of effect	RR > 2	Not possible to assess	No reason for upgrading
8. Confounding adjusted	Effect in spite of confounding working towards the nil	Not observed	No reason for upgrading
Overall Judgement			Very low quality

* Population Exposure Comparator Outcome.

Table S4. The template for assessment of quality and risk of bias of individual studies.

REFERENCE No 1

**Risk of Bias Check List for Studies on Noise and Health Outcomes**	**Study Name:** ***Audiometric Thresholds and Portable Digital Audio Player User Habits. Feder et al., 2013***	**Assessor Name: Mariola Sliwinska-Kowalska, Kamil Zaborowski**	**Date Assessed: 10.12.2014**
Study Design	Cross-sectional		
Domain	Description of criteria for judgment		Judgment of risk of bias
	Noise Exposure assessment leading to information bias<br>*Leq (32 s) and Lex(8hr) estimates. The Leq(32 s) values were taken from the two 32-s measurements for both typical and maximum settings (corrected for transfer function of the open ear). The Lex(8 h) or daily exposure level was calculated using a 3-dB exchange rate. Daily usage was estimated by dividing subjects' self-reported weekly usage in hours by seven.*		Low
	Bias due to confounding<br>*Variables included into multivariate models for audiometric frequencies and Lex(8 h)/Leq(32 s) comprised socioeconomic status, demographic factors, tubes in ears, other leisure noise exposures.*		Low
	Bias due to selection of Participants<br>237 *subjects aged 10 to 17 years. Out of 35 schools invited to participate, 19 schools declined participation. Every 3rd or 4th student on their class lists were selected and distributed equally to male and female subjects in grades 5 – 12. Low participation rate – only 11% out of 2337 invitations sent.*		Unclear
	Health outcome assessment leading to information bias I<br>*Hearing thresholds were measured by means of pure-tone audiometry.*		Low
	Health outcome assessment leading to information bias II<br>*No information is provided about blinding the health outcome and/or exposure assessment.*		Unclear
	Total Risk of Bias in Study:<br>3/5 High		

REFERENCE No 2

**Risk of Bias Check List for Studies on Noise and Health Outcomes**	**Study Name:** ***Hearing Risk Associated with the Usage of Personal Listening Devices among Urban High School Students. Sulaiman et al., 2013***	**Assessor Name: Mariola Sliwinska-Kowalska Kamil Zaborowski**	**Date Assessed: 10.12.2014**
Study Design	Cross-sectional		
Domain	Description of criteria for judgment		Judgment of risk of bias
	Noise Exposure assessment leading to information bias		

	Usual listening levels were measured while listening to initial 40 s of a pre-selected song ("Just like Heaven' by The Cure). During the test, subjects were blinded from the iPod volume setting and told to set it to their usual listening level without considering preference to the test song. Individual LAeq8h was calculated for every single person based on estimation of the level of music and hours a day of listening to the music.	Low
	Bias due to confounding<br>*Subjects with exposure to other sources of loud noises (e.g., disco, concerts, school bands, noisy tools, home stereo) at least twice per month and prior ear problems were excluded from the study. No other confounders were taken into account (e.g., head trauma, drug ototoxicity, cigarette smoking).*	Low
	Bias due to selection of Participants<br>*Subjects aged 13-16 years were recruited from three schools selected at random from the list of high schools located within 20 km of the authors' university. They had to be regular users of PLDs for the previous six months. Participants were probably not randomly selected, and the response rate was not provided. Eight out of 185 subjects were excluded due to ear abnormalities.*	Unclear
	Health outcome assessment leading to information bias I<br>*Hearing thresholds were measured by means of pure-tone audiometry and extended high frequency audiometry. Subjects were requested to refrain from listening to their PLDs for 24 h prior to the hearing test. Tinnitus was self-reported,*	Low
	Health outcome assessment leading to information bias II<br>*No information is provided about blinding the health outcome and/or exposure assessment.*	Unclear
	Total Risk of Bias in Study:<br>3/5 High	

REFERENCE No 3

**Risk of Bias Check List for Studies on Noise and Health Outcomes**	**Study Name:**<br>***Evaluation of Early Hearing Damage in Personal Listening Device Users Using Extended High-Frequency Audiometry and Otoacoustic Emissions.***<br>***Sulaiman et al., 2014***	**Assessor Name:**<br>**Mariola Sliwinska-Kowalska**<br>**Kamil Zaborowski**	**Date Assessed:**<br>**10.12.2014**
Study Design	Cross-sectional		
Domain	Description of criteria for judgment		Judgment of risk of bias
	Noise Exposure assessment leading to information bias<br>*Usual listening levels were measured while listening to initial 40 s of a pre-selected song ("Just like Heaven' by The Cure) in a quiet room (45-50 dBA ambient noise level). During the test, subjects were blinded from the iPod volume setting and told to set it to their usual listening level without considering preference to the song. Individual LAeq8h was calculated for every single person based on estimation of the level of music and hours a day listen to the music.*		Low
	Bias due to confounding		

	No prior exposure to any loud occupational noise. None of the subjects had participated in a band, was engaged in shooting activities or had a hobby of playing musical instrument. None of the subjects was exposed to other sources of loud noises (e.g., disco, concerts, noisy tools, home stereo) more than once in a month. No prior ear disorders and currently taken medications, including ototoxic drugs.	Low
	Bias due to selection of Participants	
	Subjects aged 18-30 years were recruited voluntarily from the students and staff of one university, using strictly defined inclusion and exclusion criteria. Exposed group of 35 subjects (users of PLDs for at least 1 year, 1 h/day and at >50% of the maximum volume setting) was sex- and age matched to the control group of 35 subjects who never or rarely used a PLD.	Unclear
	Health outcome assessment leading to information bias I	
	Hearing thresholds were measured by means of pure-tone audiometry and extended high frequency audiometry. TEOAE and DPOAE amplitude were measured objectively. Subjects were requested to refrain from listening to their PLDs for 24 h prior to the hearing tests.	Low
	Health outcome assessment leading to information bias II	
	No information is provided about blinding the health outcome and/or exposure assessment.	Unclear
	Total Risk of Bias in Study:	
	3/5 High	

REFERENCE No 4

**Risk of Bias Check List for Studies on Noise and Health Outcomes**	**Study Name:** ***Exposure of High School Students to Noise from Personal Music Players in Quebec City, Canada. Lévesque et al., 2010***	**Assessor Name: Mariola Sliwinska-Kowalska Kamil Zaborowski**	**Date Assessed: 10.12.2014**
Study Design	Cross-sectional		
Domain	Description of criteria for judgment		Judgment of risk of bias
	Noise Exposure assessment leading to information bias		
	Individual $L_{Aeq,8h}$ were calculated based on 2-min. measurement of music sounds listen through own PLD at "typical volume" and at "maximum level volume"; the data about the daily average length of PLD use was taken from the questionnaire .		Low
	Bias due to confounding		
	No confounding factors included		High
	Bias due to selection of Participants		
	Students aged 14-17 years were recruited voluntarily from one high school. No inclusion or exclusion criteria specified. Lack of randomization. Participation rate was 63.3%.		High
	Health outcome assessment leading to information bias I		

	No audiometric data. The presence of tinnitus was assessed by a questionnaire. Diagnostic criteria of tinnitus were not specified. It's not clear whether the outcome is a permanent tinnitus or transient tinnitus after listening to music.	High
	Health outcome assessment leading to information bias II	
	No information is provided about blinding the health outcome and/or exposure assessment.	Unclear/High
	Total Risk of Bias in Study:	
	1/5 very high	

REFERENCE No 5

**Risk of Bias Check List for Studies on Noise and Health Outcomes**	**Study Name:** ***Risky Music Listening, Permanent Tinnitus and Depression, Anxiety, Thoughts about Suicide and Adverse General Health. Vogel et al., 2014***	**Assessor Name: Mariola Sliwinska-Kowalska Kamil Zaborowski**	**Date Assessed: 10.12.2014**
Study Design	Cross-sectional		
Domain	Description of criteria for judgment		Judgment of risk of bias
	Noise Exposure assessment leading to information bias		
	No direct measurement of noise levels. Average weekly exposure time to MP3 players was estimated by referring the volume of the device to dB(A) value and multiplying days per week and hours per day to calculate (weekly) Permissible Exposure Limits (PEL_{week} = music level of 89 dBA listen for 56 h a week).		Unclear/High?
	Bias due to confounding		
	Values adjusted for age and gender		Low
	Bias due to selection of Participants		
	A total of 1228 students, aged 16-25 years of 2 Dutch secondary vocational schools were invited. No randomization. Participation rate 77.9%		Low
	Health outcome assessment leading to information bias I		
	No audiometric data. The presence of "permanent hearing-related symptoms" were categorized as "I am constantly experiencing hearing symptoms (yes, no)". There is not clear what proportion of subjects with "permanent hearing-related symptoms" experienced permanent tinnitus. Diagnostic criteria of permanent tinnitus were not specified. No validated method of tinnitus assessment.		High
	Health outcome assessment leading to information bias II		
	No information is provided about blinding the health outcome and/or exposure assessment.		Unclear/High
	Total Risk of Bias in Study:		
	2/5 High		

Table S5. The list of included and excluded studies.

**Included Studies**	
1.	Feder, K.; Marro, L.; Keith, S.E.; Michaud, D.S. Audiometric thresholds and portable digital audio player user listening habits. *Int. J. Audiol.* 2013, 52, 606-616. doi: 10.3109/14992027.2013.798687.
2.	Lévesque, B.; Fiset, R.; Isabelle, L.; Gauvin, D.; Baril, J.; Larocque, R.; Gingras, S.; Girard, S.A.; Leroux, T.; Picard, M. Exposure of high school students to noise from personal music players in Québec City, Canada. *Int. J. Child Adolescent Health* 2010, 3, 413-420.
3.	Sulaiman, A.H.; Seluakumaran, K.; Husain, R. Hearing risk associated with the usage of personal listening devices among urban high school students in Malaysia. *Public. Health* 2013, 127, 710-715.
4.	Sulaiman, A.H.; Husain, R.; Seluakumaran, K. Evaluation of early hearing damage in personal listening device users using extended high-frequency audiometry and otoacoustic emissions. *Eur. Arch. Otorhinolaryngol.* 2014, 271, 1463-1470. doi: 10.1007/s00405-013-2612-z.
5.	Vogel, I.; van de Looij-Jansen, P.M.; Mieloo, C.L.; Burdorf, A.; de Waart, F. Risky music listening, permanent tinnitus and depression, anxiety, thoughts about suicide and adverse general health. *PLoS One* 2014, 9, e98912. doi: 10.1371/journal.pone.0098912.
**Excluded Studies**	
6.	Almstedt, A.C.; Gustafsson, T.; Axelsson, A. Risk of hearing damage in connection with pop and rock concerts. The maximum permissible sound level should be legally confirmed. *Lakartidningen* 2000, 97, 1102-1104.
7.	Andrus, W.S.; Kerrigan, M.E.; Bird, K.T. Hearing in para-airport children. *Aviat. Space Environ. Med*. 1975, 46, 740-742. - airports
8.	Angrini, M.A.; Leslie, J.C. Vitamin C attenuates the physiological and behavioural changes induced by long-term exposure to noise. *Behav. Pharmacol.* 2012, 23, 119-125. doi: 10.1097/FBP.0b013e32834f9f68. – drug intervention
9.	Arch-Tirado, E.; Garnica-Escamilla, M.A.; Delgado-Hernández, A.; Campos-Munoz, T.; Rodríguez-Rodríguez, L.; Verduzco-Mendoza, A. Acoustic trauma generated by exposure to gun powder. *Cir. Cir.* 2014, 82, 528-536.
10.	Autenrieth, D.A.; Sandfort, D.R.; Lipsey, T.; Brazile, W.J. Occupational exposures to noise resulting from the workplace use of personal media players at a manufacturing facility. *J. Occup. Environ. Hyg.* 2012, 9, 592-601.
11.	Axelsson, A. Recreational exposure to noise and its effects. *Noise Control Eng J.* 1996, 44, 127-134.
12.	Axelsson, A.; Rosenhall, U.; Zachau, G. Hearing in 18-year-old Swedish males. *Scand. Audiol*. 1994, 23, 129-134.
13.	Axelsson, A.; Andersson, S.; Gu, L.D. Acupuncture in the management of tinnitus: a placebo-controlled study. *Audiology* 1994, 33, 351-360.
14.	Axelsson, A.; Dengerink, H.; Hellström, P.A.; Mossberg, A.M. The sound world of the child. The relationship between daily activities and hearing acuity. *Scand. Audiol.* 1993, 22, 117-124.
15.	Axelsson, A.; Hellström, P.A.; Altschuler, R.; Miller, J.M. Inner ear damage from toy cap pistols and fire-crackers. *Int. J. Pediatr. Otorhinolaryngol.* 1991, 21, 143-148.
16.	Banerjee, D. Research on road traffic noise and human health in India: Review of literature from 1991 to current. *Noise Health* 2012, 14, 113-118. doi: 10.4103/1463-1741.97255.
17.	Barcelos, D.D.;, Dazzi, N.S. Efeitos do mp3 player na audiçao. *Rev. CEFAC*. 2014, 16, 779-792.
18.	Barlow, C. Potential hazard of hearing damage to students in undergraduate popular music courses. *Med. Probl. Perform. Art.* 2010, 25, 175-182.
19.	Basner, M.; Babisch, W.; Davis, A.; Brink, M.; Clark, C.; Janssen, S.; Stansfeld, S. Auditory and non-auditory effects of noise on health. *Lancet* 2014, 383,1325-1332. doi: 10.1016/S0140-6736(13)61613-X.
20.	Basner, M.; Brink, M.; Bristow, A.; de Kluizenaar, Y.; Finegold, L.; Hong, J.; Janssen, S.A.; Klaeboe, R.; Leroux, T.; Lieb, A.; Matsui, T.; Schwela, D.; Sliwinska-Kowalska, M.; Sörqvist, P. ICBEN review of research on the biological effects of noise 2011-2014. *Noise Health* 2015, 17, 57-82. doi: 10.4103/1463-1741.153373.
21.	Beach, E.; Williams, W.; Gilliver, M. Estimating young Australian adults' risk of hearing damage from selected leisure activities. *Ear Hear*. 2013, 34, 75-82. doi: 10.1097/AUD.0b013e318262ac6c.
22.	Beach, E.F.; Gilliver, M.; Williams, W. A snapshot of young adults' noise exposure reveals evidence of 'Binge Listening'. *Appl. Acoust.* 2014, 77, 71–75.
23.	Berg AL, Serpanos YC. High frequency hearing sensitivity in adolescent females of a lower socioeconomic status over a period of 24 years (1985-2008). *J. Adolesc. Health* 2011, 48, 203-208. doi: 10.1016/j.jadohealth.2010.06.014.
24.	Berg, R.L.; Pickett, W.; Randolph, M.F.; Broste, S.K.; Knobloch, M.J.; Wood, D.J.; Kirkhorn, S.R.; Linnemana, J.G.; Marlenga, B. Hearing conservation program for agricultural students: Short-term outcomes from a cluster-randomized trial with planned long-term follow-up. *Prev. Med.* 2009, 49, 546–552.
25.	Berger, E.H.; Megerson, S.C.; Stergar, M.E. Personal music players: Are we measuring the sound levels correctly? *ASHA Lead* 2009, 14, 14–17. doi:10.1044/leader.FTR6.14102009.14.
26.	Bhagat, S.P.; Davis, A.M. Modification of otoacoustic emissions following ear-level exposure to MP3 player music. *Int. J. Audiol.* 2008, 47, 751-760. doi: 10.1080/14992020802310879.
27.	Biassoni, E.C.; Serra, M.R.; Hinalaf, M.; Abraham, M.; Pavlik, M.; Villalobo, J.P.; Curet, C.; Joekes, S.; Yacci, M.R.; Righetti, A. Hearing and loud music exposure in a group of adolescents at the ages of 14-15 and retested at 17-18. *Noise Health* 2014, 16, 331-341. doi: 10.4103/1463-1741.140515.
28.	Biassoni, E.C.; Serra, M.R.; Richtert, U.; Joekes, S.; Yacci, M.R.; Carignani, J.A.; Abraham, S.; Minoldo, G.; Franco, G. Recreational noise exposure and its effects on the hearing of adolescents. Part II: development of hearing disorders. *Int. J. Audiol*. 2005, 44, 74-85.

29. Bogoch, I.I.; House, R.A.; Kudla, I. Perceptions about hearing protection and noise-induced hearing loss of attendees of rock concerts. *Can. J. Public. Health* 2005, 96,69-72.
30. Bohlin, M.C.; Erlandsson, S.I. Risk behaviour and noise exposure among adolescents. *Noise Health* 2007, 9, 55-63.
31. Borchgrevink, H.M. Does health promotion work in relation to noise? *Noise Health* 2003, 5, 25-30.
32. Bouccara, D.; Ferrary, E.; Sterkers, O. Effects of noise on inner ear. *Med. Sci*. 2006, 22, 979-984.
33. Breinbauer, K.H.A.; Anabalón, B.; Gutierrez, D.; Cárcamo, R.; Olivares, C.; Caro, J. Output capabilities of personal music players and assessment of preferred listening levels of test subjects: outlining recommendations for preventing music-induced hearing loss. *Laryngoscope* 2012, 122,2549-2556.
34. Breinbauer, K.H.A.; Anabalón, B. Reproductores de música personal: Una conducta de riesgo emergente. *Rev. Otorrinolaringol. Cir. Cabeza Cuello.* 2009, 69, 213-220.
35. Breinbauer, K.H.A.; Anabalón, B.J.L.; Gutiérrez, C.D.; Caro L.J. Estimación de riesgos y hábitos de uso de reproductores de música personal en una muestra de población chilena. *Rev. Otorrinolaringol. Cir. Cabeza Cuello* 2011, 71, 31-38.
36. Budimčić, M.; Seke, K.; Krsmanović, S.; Živić, L. Auditory risk behaviours and hearing problems among college students in Serbia. *Med Glas* (Zenica). 2014, 11,361-366.
37. Carter, L.; Williams, W.; Black, D.; Bundy, A. The leisure-noise dilemma: hearing loss or hearsay? What does the literature tell us? *Ear Hear.* 2014, 35, 491-505. doi: 10.1097/01.aud.0000451498.92871.20.
38. Cassano, E.; Bavaro, P.; Aloise, I.; Bobbio, E.; Renna, M. Music through earphones: an underestimated risk. *Med. Lav.* 2008, 99, 362-365.
39. Chen, T.; Chen, S. Effects of aircraft noise on hearing and auditory pathway function of school-age children. *Int. Arch. Occup. Environ. Health* 1993, 65, 107-111.- airport
40. Chung, J.H.; des Roches, C.M.; Meunier, J.; Eavey, R.D. Evaluation of noise-induced hearing loss in young people using a web-based survey technique. *Pediatrics* 2005, 115, 861-867.
41. Clark, W.W. Hearing: the effects of noise. *Otolaryngol. Head Neck Surg.* 1992, 106, 669-676.
42. Cone, B.K.; Wake, M.; Tobin, S.; Poulakis, Z.; Rickards, F.W. Slight-mild sensorineural hearing loss in children: audiometric, clinical, and risk factor profiles. *Ear Hear.* 2010, 31, 202-212. doi: 10.1097/AUD.0b013e3181c62263.
43. Cranston, C.J.; Brazile, W.J.; Sandfort, D.R.; Gotshall, R.W. Occupational and recreational noise exposure from indoor arena hockey games. J. Occup. Environ. Hyg. 2013, 10, 11-16. doi: 10.1080/15459624.2012.736341.
44. Dalton, D.S.; Cruickshanks, K.J.; Wiley, T.L.; Klein, B.E.; Klein, R.; Tweed, T.S. Association of leisure-time noise exposure and hearing loss. *Audiology* 2001, 40, 1–9.
45. Danhauer, J.L.; Johnson, C.E.; Byrd, A.; DeGood, L.; Meuel, C.; Pecile, A.; Koch, L.L. Survey of college students on iPod use and hearing health. *J. Am. Acad. Audiol.* 2009, 20, 5-27.
46. Danhauer, J.L.; Johnson, C.E.; Dunne, A.F.; Young, M.D.; Rotan, S.N.; Snelson, T.A.; Stockwell, J.S.; McLain, M.J. Survey of high school students' perceptions about their iPod use, knowledge of hearing health, and need for education. *Lang Speech Hear. Ser.* 2012, 43, 14-35. doi: 10.1044/0161-1461(2011/10-0088).
47. Derebery, M.J.; Vermiglio, A.; Berliner, K.I.; Potthoff, M.; Holguin, K. Facing the music: pre- and postconcert assessment of hearing in teenagers. *Otol. Neurotol.* 2012, 33, 1136-1141. doi: 10.1097/MAO.0b013e31825f2328.
48. Drake-Lee, A.B. Beyond music: auditory temporary threshold shift in rock musicians after a heavy metal concert. *J. R. Soc. Med.* 1992, 85, 617-619.
49. Dudarewicz, A.; Pawlaczyk-Luszczynska, M.; Sliwinska-Kowalska, M. Developing the method for assessing non-occupational exposure to noise. *Med. Pr.* 2007, 58, 231-242.
50. Eggemann, C.; Koester, M.; Zorowka, P. Hearing loss due to leisure time noise is on the rise. The ear also needs a rest period. *MMW Fortschr. Med.* 2002, 144, 30-33.
51. Einhorn, R. Observations from a musician with hearing loss. *Trends Amplif.* 2012, 16, 179-182. doi: 10.1177/1084713812468513.
52. Engard, D.J.; Sandfort, D.R.; Gotshall, R.W.; Brazile, W.J. Noise exposure, characterization, and comparison of three football stadiums. *J. Occup. Environ. Hyg*. 2010, 7, 616-21. doi: 10.1080/15459624.2010.510107.
53. England, B.; Larsen, J.B. Noise levels among spectators at an intercollegiate sporting event. *Am. J. Audiol.* 2014, 23, 71-78. doi: 10.1044/1059-0889(2013/12-0071).
54. Epstein, M.; Marozeau, J.; Cleveland, S. Listening habits of iPod users. *J. Speech Lang. Hear. R.* 2010, 53, 1472-1477. doi: 10.1044/1092-4388(2010/09-0059).
55. Eysel-Gosepath, K.; Pape, H.G.; Erren, T.; Thinschmidt, M.; Lehmacher, W.; Piekarski, C. Sound levels in nursery schools. *HNO* 2010, 58, 1013-1020. doi: 10.1007/s00106-010-2121-y.
56. Fallon, L.F. Hearing loss and music: Report of research and an illustrative case study. *J. Controversial Med. Claims* 2010, 17, 17–20.
57. Figueiredo, R.R.; Azevedo, A.A.; Oliveira, P.M.; Amorim, S.P.; Rios, A.G.; Baptista, V. Incidence of tinnitus in mp3 player users. *Braz. J. Otorhinolaryngol.* 2011, 77, 293-298.
58. Fisch, L. Aircraft noise and hearing impairment in children. *Br. J. Audiol.* 1981, 15, 231-240. – airports
59. Flamme, G.A.; Liebe, K.; Wong, A. Estimates of the auditory risk from outdoor impulse noise. I: Firecrackers. *Noise Health* 2009, 11, 223-230. doi: 10.4103/1463-1741.56216.
60. Fleischer, G.; Hoffmann, E.; Lang, R.; Müller, R. Documentation of the effects of child cap pistols. *HNO* 1999, 47, 535-540.
61. Fleischer, G.; Hoffmann, E.; Müller, R.; Lang, R. Toy cap pistols and their effect on hearing. *HNO* 1998, 46, 815-820.
62. Fleming, C. Assessment of noise exposure level of bar staff in discotheques. Applied Acoustics. 1996 Sep;49(1):85-94. – occupational exposure

63. Fligor, B.J. Personal listening devices and hearing loss: seeking evidence of a long term problem through a successful short-term investigation. *Noise Health*. 2009, 11, 129-131. doi: 10.4103/1463-1741.53356.
64. Fuentes, L.E.; Cardemil, M.F. Validation of criteria and construct for the creation of a questionnaire on noise exposure. *Rev. Otorrinolaringol. Cir. Cabeza Cuello* 2014, 74, 21-30.
65. Gershon, R.R.; Sherman, M.F.; Magda, L.A.; Riley, H.E.; McAlexander, T.P.; Neitzel, R. Mass transit ridership and self-reported hearing health in an urban population. *J. Urban Health* 2013, 90, 262-275. doi: 10.1007/s11524-012-9734-2.
66. Gilles, A.; De Ridder, D.; Van Hal, G.; Wouters, K.; Kleine Punte, A.; Van de Heyning, P. Prevalence of leisure noise-induced tinnitus and the attitude toward noise in university students. *Otol. Neurotol.* 2012, 33, 899-906. doi: 10.1097 /MAO.0b013e31825d640a.
67. Gilles, A.; Van Hal, G.; De Ridder, D.; Wouters, K.; Van de Heyning, P. Epidemiology of noise-induced tinnitus and the attitudes and beliefs towards noise and hearing protection in adolescents. *PLoS One* 2013, 24, 8, e70297. doi: 10.1371/journal.pone.0070297.
68. Gilles, A.; Paul Vde, H. Effectiveness of a preventive campaign for noise-induced hearing damage in adolescents. *Int. J. Pediatr. Otorhinolaryngol.* 2014, 78, 604-609. doi: 10.1016/j.ijporl.2014.01.009.
69. Gilles, A.; Thuy, I.; De Rycke, E.; Van de Heyning, P. A little bit less would be great: adolescents' opinion towards music levels. *Noise Health* 2014, 16, 285-291. doi: 10.4103/1463-1741.140508.
70. Gilliver, M.; Carter, L.; Macoun, D.; Rosen, J.; Williams, W. Music to whose ears? The effect of social norms on young people's risk perceptions of hearing damage resulting from their music listening behavior. *Noise Health* 2012, 14, 47-51. doi: 10.4103/1463-1741.95131.
71. Göthe, C.J.; Cynkier, I.; Lind, M.L.; Blomberg, R.; Svensson, E.B.; Ytterlind, A. Exposure to noise by electronic amplified music-rock concerts carry a risk of hearing damage. *Lakartidningen* 1992, 89, 3579-3580.
72. Green, K.B.; Pasternack, B.S.; Shore, R.E. Effects of aircraft noise on hearing ability of school-age children. *Arch. Environ. Health* 1982, 37, 284-289. - airport
73. Guoqing, D.; Xiaoyi, L.; Xiang, S.; Zhengguang, L.; Qili, L. Investigation of the relationship between aircraft noise and community annoyance in China. *Noise Health* 2012, 14, 52-57. doi: 10.4103/1463-1741.95132. – non auditory effects
74. Gupta, D. Vishwakarma, S.K. Toy weapons and firecrackers: a source of hearing loss. *Laryngoscope* 1989, 99, 330-334.
75. Hammer, M.S.; Swinburn, T.K.; Neitzel, R.L. Environmental noise pollution in the United States: developing an effective public health response. *Environ. Health Persp.* 2014, 122, 115-119. doi: 10.1289/ehp.1307272.
76. Hannah, K.; Ingeborg, D.; Leen, M.; Annelies, B.; Birgit, P.; Freya, S.; Bart, V. Evaluation of the olivocochlear efferent reflex strength in the susceptibility to temporary hearing deterioration after music exposure in young adults. *Noise Health* 2014, 16, 108-115. doi: 10.4103/1463-1741.132094.
77. Harrison, R.V. Noise-induced hearing loss in children: A 'less than silent' environmental danger. *Paediatr. Child Health* 2008, 13, 377-382.
78. Harrison, R.V. The prevention of noise induced hearing loss in children. *Int. J. Pediatr.* 2012, 2012, 1-13. doi: 10.1155/2012/473541. Epub 2012 Dec 13.
79. Häusler, R. The effects of acoustic overstimulation. *Ther. Umsch*. 2004, 61, 21-29.
80. Hellstrom, P.A.; Dengerink, H.A.; Axelsson, A. Noise levels from toys and recreational articles for children and teenagers. *Br. J. Audiol.* 1992, 26, 267-270.
81. Hendershot, C.; Pakulski, L.A.; Thompson, A.; Dowling, J.; Price, J.H. School nurses' role in identifying and referring children at risk of noise-induced hearing loss. *J. Sch. Nurs.* 2011, 27, 380-389. doi: 10.1177/1059840511411716.
82. Henderson, E.; Testa, M.A.; Hartnick, C. Prevalence of noise-induced hearing-threshold shifts and hearing loss among US youths. *Pediatrics* 2011, 127, e39-46.
83. Henry, P.; Foots, A. Comparison of user volume control settings for portable music players with three earphone configurations in quiet and noisy environments. *J. Am. Acad. Audiol*. 2012, 23, 182-191.
84. Hodgetts, W.E.; Rieger, J.M.; Szarko, R.A. The Effects of Listening Environment and Earphone Style on Preferred Listening Levels of Normal Hearing Adults Using an MP3 Player. *Ear Hear.* 2007, 28, 290-297.
85. Hohmann, B.W.; Mercier, V.; Felchlin, I. Effects on hearing caused by personal cassette players, concerts, and discotheques and conclusions for hearing conservation in Switzerland. *Noise Control Eng. J.* 1999, 47, 163-165.
86. Holgers, K.M.; Pettersson, B. Noise exposure and subjective hearing symptoms among school children in Sweden. *Noise Health* 2005, 7, 27-37.
87. Hoover, A.; Krishnamurti, S. Survey of college students' MP3 listening: Habits, safety issues, attitudes, and education. *Am. J. Audiol.* 2010, 19, 73-83.
88. Hutchinson Marron, K.; Marchiondo, K.; Stephenson, S.; Wagner, S.; Cramer, I.; Wharton, T.; Hughes, M.; Sproat, B.; Alessio, H. College students' personal listening device usage and knowledge. *Int. J. Audiol.* 2015, 54, 384-390. doi:10.3109/14992027.2014.986691.
89. Hutchinson Marron, K.; Sproat, B.; Ross, D.; Wagner, S.; Alessio, H. Music listening behavior, health, hearing and otoacoustic emission levels. *Int. J. Environ. Res. Public Health* 2014, 11, 7592-7607. doi: 10.3390/ijerph110807592.
90. Iheanacho, I. Listen up: deafness and personal audio players. *BMJ* 2010, 341, c3539. doi: 10.1136/bmj.c3539.
91. Ivory, R.; Kane, R.; Diaz, R.C. Noise-induced hearing loss: a recreational noise perspective. *Curr. Opin. Otolaryngol*. 2014, 22, 394-398. doi: 10.1097/MOO.0000000000000085.
92. Jacobson, G. Portable media players, preferred listening levels, earphones, and background noise. *J. Am. Acad. Audiol.* 2012, 23, 144. doi: 10.3766/jaaa.23.3.1.
93. Jadid, K.; Klein, U.; Meinke, D. Assessment of noise exposures in a pediatric dentistry residency clinic. *Pediatr. Dent.* 2011, 33, 343-348. – occupational setting

94. Job, A.; Raynal, M.; Tricoire, A.; Signoret, J.; Rondet, P. Hearing status of French youth aged from 18 to 24 years in 1997: a cross-sectional epidemiological study in the selection centres of the army in Vincennes and Lyon. *Rev. Epidemiol. Sante* 2000, 48, 227-237.
95. Jofré, P.D.; De la Paz, P.F.; Platzer, M.L.; Anabalón, B.J.L.; Grasset, E.E.; Barnafi, R.N. Evaluación de la exposición a ruido social en jóvenes chilenos. *Rev. Otorrinolaringol. Cir. Cabeza Cuello* 2009, 69, 23-28.
96. Johnson, O.; Andrew, B.; Walker, D.; Morgan, S.; Aldren, A. British university students' attitudes towards noise-induced hearing loss caused by nightclub attendance. *J. Laryngol. Otol.* 2014, 128(1):29-34. doi: 10.1017/S0022215113003241.
97. Jokitulppo, J.S.; Björk, E.A.; Akaan-Penttilä, E. Estimated leisure noise exposure and hearing symptoms in Finnish teenagers. *Scand. Audiol.* 1997, 26, 257-262.
98. Kardous, C.A.; Morata, T.C. Occupational and recreational noise exposures at stock car racing circuits:An exploratory survey of three professional race tracks. *Noise Control Eng. J.* 2010, 58, 54.
99. Keith, S.E.; Michaud, D.S.; Feder, K.; Haider, I.; Marro, L.; Thompson, E.; Marcoux, A.M. MP3 player listening sound pressure levels among 10 to 17 year old students. *J. Acoust. Soc. Am.* 2011, 130, 2756-2764.
100. Kelly, A.C.; Boyd, S.M.; Henehan, G.T.; Chambers, G. Occupational noise exposure of nightclub bar employees in Ireland. *Noise Health* 2012, 14, 148-154. doi: 10.4103/1463-1741.99868. – occupational exposure
101. Keppler, H.; Dhooge, I.; Maes, L.; D'haenens, W.; Bockstael, A.; Philips, B.; Swinnen, F.; Vinck, B. Noise-Induced Hearing Loss From MP3 Players reply. *Arch. Otolaryngol.* 2010, 136, 1280.
102. Keppler, H.; Dhooge, I.; Maes, L.; D'haenens, W.; Bockstael, A.; Philips, B.; Swinnen, F.; Vinck, B. Short-term auditory effects of listening to an MP3 player. *Arch. Otolaryngol.* 2010, 136, 538-548. doi: 10.1001/archoto.2010.84.
103. Khatter, K. Personal music players and hearing loss: are we deaf to the risks? *Open Med.* 2011, 5, e137-8.
104. Kim, E.J. Analysis of Factors Affecting Output Levels and Frequencies of MP3 Players. *Korean J. Audiol*. 2013, 17, 59-64. doi: 10.7874/kja.2013.17.2.59.
105. Kim, E.J. Problems of Usage of Earphones, PCPs on Hearing in adolescents. *J. Korean Soc. Sch. Health* 2009, 107-118.
106. Kim, M.G.; Hong, S.M.; Shim, H.J.; Kim, Y.D.; Cha, C.I.; Yeo, S.G. Hearing threshold of Korean adolescents associated with the use of personal music players. *Yonsei Med. J.* 2009, 50, 771-776. doi: 10.3349/ymj.2009.50.6.771.
107. Knobel, K.A.; Lima, M.C. Knowledge, habits, preferences, and protective behavior in relation to loud sound exposures among Brazilian children. *Int. J. Audiol.* 2012, 51, 12-19. doi: 10.3109/14992027.2011.637307.
108. Kramer, M.B.; Wood, D. Noise-induced hearing loss in rural schoolchildren. *Scand. Audiol.* 1982, 11, 279-280.
109. Lamm, K.; Michaelis, C.; Deingruber, K.; Scheler, R.; Steinhoff, H.J.; Gröber, I.; Huth, M.; Kutscher, C.; Arnold, W. Inner ear damage due to leisure and broadband noise. An experimental study on initial and permanent functional and morphological damage. *HNO* 2004, 52, 301-310.
110. Lao, X.Q.; Yu, I.T.; Au, D.K.; Chiu, Y.L.; Wong, C.C.; Wong, T.W. Noise exposure and hearing impairment among Chinese restaurant workers and entertainment employees in Hong Kong. *PLoS One* 2013, 15, e70674. doi: 10.1371/journal.pone.0070674. - ccupational exposure
111. Le Prell, C.G.; Dell, S.; Hensley, B.; Hall, J.W. 3rd; Campbell, K.C.; Antonelli, P.J.; Green, G.E.; Miller, J.M.; Guire, K. Digital music exposure reliably induces temporary threshold shift in normal-hearing human subjects. *Ear Hear.* 2012, 33, e44-58. doi: 10.1097/AUD.0b013e31825f9d89.
112. Le Prell, C.G.; Hensley, B.N.; Campbell, K.C.; Hall, J.W. 3rd; Guire, K. Evidence of hearing loss in a 'normally-hearing' college-student population. *Int. J. Audiol.* 2011, 50, 21-31. doi: 10.3109/14992027.2010.540722.
113. Le Prell, C.G.; Spankovich, C.; Lobarinas, E.; Griffiths, S.K. Extended high-frequency thresholds in college students: effects of music player use and other recreational noise. *J. Am. Acad. Audiol.* 2013, 24, 725-739. doi: 10.3766/jaaa.24.8.9.
114. Le Prell, C.G.; Yang, Q.; Harris, J.G. Modification of digital music files for use in human temporary threshold shift studies. *J. Acoust. Soc. Am.* 2011, 130, EL142-146. doi: 10.1121/1.3630017.
115. Lee, G.J.; Lim, M.Y.; Kuan, A.Y.; Teo, J.H.; Tan, H.G.; Low, W.K. The music listening preferences and habits of youths in Singapore and its relation to leisure noise-induced hearing loss. *Singapore Med. J.* 2014, 55, 72-77.
116. Lepore, S.J.; Shejwal, B.; Kim, B.H.; Evans, G.W. Associations between chronic community noise exposure and blood pressure at rest and during acute noise and non-noise stressors among urban school children in India. *Int. J. Environ. Res. Public Health* 2010, 7, 3457-3466. doi: 10.3390/ijerph7093457. – non auditory effects.
117. Leroux, T.; Laroche, C. Regulating the Noise-level of toys for children in Canada. *J. De Phys. IV* 1992, 241-244.
118. Levey, S.; Fligor, B.J.; Cutler, C.; Harushimana, I. Portable music player users: cultural differences and potential dangers. *Noise Health* 2013, 15, 296-300. doi: 10.4103/1463-1741.116553.
119. Levey, S.; Levey, T.; Fligor, B.J. Noise exposure estimates of urban MP3 player users. *J Speech Lang. Hear. Res.* 2011, 54, 263-277.
120. Lewis, R.C.; Gershon, R.R.; Neitzel, R.L. Estimation of permanent noise-induced hearing loss in an urban setting. *Environ. Sci. Technol.* 2013, 47, 6393-6399. doi: 10.1021/es305161z.
121. Li, L.P.; Chuang, A.Y.; McMahon, C.; Tung, T.H.; Chen, J.K. Low body mass index and jaw movement are protective of hearing in users of personal listening devices. *Laryngoscope* 2013, 123, 1983-1987. doi: 10.1002/lary.23955.
122. Liang, M.; Zhao, F.; French, D.; Zheng, Y. Characteristics of noise-canceling headphones to reduce the hearing hazard for MP3 users. *J. Acoust. Soc. Am.* 2012, 131, 4526-4534.
123. Long, S.S. Practitioners, parents, and users of MP3 players listen up! *J. Pediatr*. 2008, 152, A1. doi: 10.1016/j.jpeds.2008.01.008.
124. Maffei, L.; Masullo, M.; Palmieri, U. Noise exposure for personal music player users in metros. *Noise Control Eng. J.* 2011, 59, 559-567.

125. Mahboubi, H.; Oliaei, S.; Badran, K.W.; Ziai, K.; Chang, J.; Zardouz, S.; Shahriari, S.; Djalilian, H.R. Systematic assessment of noise amplitude generated by toys intended for young children. *Otolaryngol Head Neck Surg.* 2013, 148, 1043-1047. doi: 10.1177/0194599813482293.
126. Maia, J.R.; Russo, I.C. Study of the hearing of rock and roll musicians. *Pro Fono.* 2008, 20, 49-54.
127. Marmut, Z.; Belojevic, G.; Backovic, D.; Zivojinovic, J.I.; Tomanic, M.; Hadzic, E. Tinnitus among Serbian secondary school students in relation to their behavior and habits. *Noise Health* 2014, 16, 73-78. doi: 10.4103/1463-1741.132080.
128. Marron, K.H.; Sproat, B.; Ross, D.; Wagner, S.; Alessio, H. Music listening behavior, health, hearing and otoacoustic emission levels. *Int. J. Environ. Res. Public Health* 2014, 11, 7592-7607. doi: 10.3390/ijerph110807592.
129. Martínez-Wbaldo Mdel, C.; Soto-Vázquez, C.; Ferre-Calacich, I.; Zambrano-Sánchez, E.; Noguez-Trejo, L.; Poblano, A. Sensorineural hearing loss in high school teenagers in Mexico City and its relationship with recreational noise. *Cad. Saude Publica* 2009, 25, 2553-2561.
130. McLaren, S.J.; Page, W.H.; Parker, L.; Rushton, M. Noise producing toys and the efficacy of product standard criteria to protect health and education outcomes. *Int. J. Environ. Res. Public Health* 2013, 11, 47-66. doi: 10.3390/ijerph110100047.
131. McNeill, K.; Keith, S.E.; Feder, K.; Konkle, A.T.; Michaud, D.S. MP3 player listening habits of 17 to 23 year old university students. *J. Acoust. Soc. Am.* 2010, 128, 646-653. doi: 10.1121/1.3458853.
132. Mercier, V.; Luy, D.; Hohmann, B.W. The sound exposure of the audience at a music festival. *Noise Health* 2003, 5, 51-58.
133. Metternich, F.U.; Brusis, T. Acute hearing loss and tinnitus related to strongly amplified music. *Laryngorhinootologie* 1999, 78, 614-619
134. Meyer, R.E.; Schwab, C.V.; Bern, C.J. Tractor Noise Exposure Levels for Bean-bar Riders. *Transactions of the ASAE.* 1993, 36, 1049-1056. – occupational exposure
135. Meyer-Bisch, C. Measuring noise. *Med Sci* 2005, 21, 546-550.
136. Miller, V.L.; Stewart, M.; Lehman, M. Noise exposure levels for student musicians. *Med. Probl. Perform. Ar.* 2007, 22, 160-164.
137. Mora-Magana, I.; Collado-Corona, M.A.; Toral-Martinon, R.; Cano, A. Acoustic trauma caused by lightning. *Int. J. Pediatr. Otorhinolaryngol*. 1996, 35, 59-69.
138. Morioka, I.; Luo, W.Z.; Miyashita, K.; Takeda, S.; Wang, Y.X.; Li, S.C. Hearing impairment among young Chinese in a rural area. *Public Health* 1996, 110, 293-297.
139. Mostafapour, S.P.; Lahargoue, K.; Gates, G.A. Noise-induced hearing loss in young adults: the role of personal listening devices and other sources of leisure noise. *Laryngoscope* 1998, 108, 1832-1839.
140. Muchnik, C.; Amir, N.; Shabtai, E.; Kaplan-Neeman, R. Preferred listening levels of personal listening devices in young teenagers: self reports and physical measurements. *Int. J. Audiol*. 2012, 51, 287-293.
141. Neitzel, R.L.; Gershon, R.R.; McAlexander, T.P.; Magda, L.A.; Pearson, J.M. Exposures to transit and other sources of noise among New York City residents. *Environ. Sci. Technol.* 2012, 46, 500-508. doi: 10.1021/es2025406.
142. Nondahl, D.M.; Cruickshanks, K.J.; Wiley, T.L.; Klein, R.; Klein, B.E.; Tweed, T.S. Recreational firearm use and hearing loss. *Arch. Fam. Med.* 2000, 9, 352-357.
143. Nottet, J.B.; Truy, E. Prevention and management of acoustic traumas. *La Revue du praticien* 2009, 59, 632-638.
144. Ono, H.; Deguchi, T.; Ino, T.; Okamoto, K.; Takyu, H. The level of the musical loud sound and noise induced hearing impairment. *J. UOEH*. 1986, 20, 151-61.
145. Pandya, B.H.; Settles, G.S.; Miller, J.D. Schlieren imaging of shock waves from a trumpet. *J. Acoust. Soc. Am.* 2003, 114, 3363-3367.
146. Pawlaczyk-Luszczynska, M.; Dudarewicz, A.; Zaborowski, K.; Zamojska, M.; Sliwinska-Kowalska, M. Noise induced hearing loss: Research in central, eastern and south-eastern Europe and newly independent states. *Noise Health* 2013, 15, 55-66.
147. Pellegrino, E.; Lorini, C.; Allodi, G.; Buonamici, C.; Garofalo, G.; Bonaccorsi, G. Music-listening habits with MP3 player in a group of adolescents: a descriptive survey. *Ann. Ig.* 2013, 25, 367-376.
148. Peng, J.H.; Tao, Z.Z.; Huang, Z.W. Risk of Damage to Hearing from Personal Listening Devices in Young Adults. *J. Otolaryngol.* 2007, 36, 181-185.
149. Petrescu, N. Loud music listening. *Mcgill J. Med.* 2008, 11, 169-176.
150. Pfeiffer, M.; Rocha, R.L.; Oliveira, F.R.; Frota, S. Intercorrencia audiológica em músicas após um show de rock. *Rev. CEFAC.* 2007, 423-429.
151. Portnuff, C.D.; Fligor, B.J.; Arehart, K.H. Self-report and long-term field measures of MP3 player use: how accurate is self-report? *Int. J. Audiol.* 2013, 52, 33-40.
152. Portnuff, C.D.; Fligor, B.J.; Arehart, K.H. Teenage use of portable listening devices: a hazard to hearing? *J. Am. Acad. Audiol.* 2011, 22, 663-677.
153. Portnuff, C.D.; Fligor, B.J.; Arehart, K.H. New Measurement Techniques for Portable Listening Devices: Technical Report. *J. Audio Eng. Soc.* 2013, 61, 749-754.
154. Potegal, M.; Yund, B.; Rudser, K.; Ahmed, A.; Delaney, K.; Nestrasil, I.; Whitley, C.B.; Shapiro, E.G. Mucopolysaccharidosis Type IIIA presents as a variant of Klüver-Bucy syndrome. *J. Clin. Exp. Neuropsychol.* 2013, 35, 608-616. doi: 10.1080/13803395.2013.804035.
155. Punch, J.L.; Elfenbein, J.L.; James, R.R. Targeting hearing health messages for users of personal listening devices. *Am. J. Audiol.* 2011. 20, 69-82. doi: 10.1044/1059-0889(2011/10-0039).
156. Rabinowitz, P.M. Hearing loss and personal music players. *BMJ* 2010, 20, 340:c1261. doi: 10.1136/bmj.c1261.

157. Rekha, T.; Unnikrishnan, B.; Mithra, P.P.; Kumar, N.; Bukelo, M.J.; Ballala, K. Perceptions and practices regarding use of personal listening devices among medical students in coastal south India. *Noise Health* 2011, 13, 329-332. doi: 10.4103/1463-1741.85500.
158. Rose, A.S.; Ebert, C.S.; Prazma, J.; Pillsbury, H.C. Noise exposure levels in stock car auto racing. *Ear. Nose. Throat. J.* 2008, 87, 689-692.
159. Royer, R.D. Sound pressure levels and frequencies in secondary public school band programs. *J. Band Res.* 2003, 38, 22-43.
160. Royster, J.D.; Royster, L.H.; Killion, M.C. Sound exposures and hearing thresholds of symphony orchestra musicians. *J. Acoust. Soc. Am.* 1991, 89, 2793-2803.
161. Ryberg, J.B. A national project to evaluate and reduce high sound pressure levels from music. *Noise Health* 2009, 11, 124-128. doi: 10.4103/1463-1741.50698.
162. Sadhra, S.; Jackson, C.A.; Ryder, T.; Brown, M.J. Noise exposure and hearing loss among student employees working in university entertainment venues. *Ann. Occup. Hyg.* 2002, 46, 455-463.
163. European Commission - Scientific Committee on Emerging and Newly Identified Health Risks. Potential health risks of exposure to noise from personal music players and mobile phones including a music playing function. 2008. Available online: http://ec.europa.eu/health/ph_risk/committees/04_scenihr/docs/scenihr_o_018.pdf (accessed on 10 October 2016).
164. Sandell, J.; Berntson, A.; Sjoesten, P.; Blomgren, G.; Kaehaeri, K. Acoustic intervention in a live music club. 2007, 93, 843-849.
165. Santaolalla Montoya, F.; Ibargüen, A.M.; Vences, A.R.; del Rey, A.S.; Fernandez, J.M. Evaluation of cochlear function in normal-hearing young adults exposed to MP3 player noise by analyzing transient evoked otoacoustic emissions and distortion products. *J. Otolaryngol. Head Neck Surg.* 2008, 37, 718-724.
166. Schmidt, J.H.; Pedersen, E.R.; Juhl, P.M.; Christensen-Dalsgaard, J.; Andersen, T.D.; Poulsen, T.; Balum, J. Sound exposure of symphony orchestra musicians. *Ann. Occup. Hyg.* 2011, 55, 893-905. doi: 10.1093/annhyg/mer055.
167. Schupp, K.; Childers, W.A. Hearing loss in adolescents. Portable music players present a new danger. *Adv. NPs. PAs.* 2012, 3, 24-26.
168. Segal, S.; Eviatar, E.; Lapinsky, J.; Shlamkovitch, N.; Kessler, A. Inner ear damage in children due to noise exposure from toy cap pistols and firecrackers: a retrospective review of 53 cases. *Noise Health* 2003, 5, 13-18.
169. Sekhar, D.L.; Rhoades, J.A.; Longenecker, A.L.; Beiler, J.S.; King, T.S.; Widome, M.D.; Paul, I.M. Improving detection of adolescent hearing loss. *Arch. Pediatr. Adolesc. Med.* 2011, 165, 1094-1100. doi: 10.1001/archpediatrics.2011.188.
170. Serra, M.R.; Biassoni, E.C.; Hinalaf, M.; Abraham, M.; Pavlik, M.; Villalobo, J.P.; Curet, C.; Joekes, S,; Yacci, M.R.; Righetti, A. Hearing and loud music exposure in 14-15 years old adolescents. *Noise Health* 2014, 16, 320-330. doi: 10.4103/1463-1741.140512.
171. Shah, S.; Gopal, B.; Reis, J.; Novak, M. Hear today, gone tomorrow: an assessment of portable entertainment player use and hearing acuity in a community sample. *J. Am. Board Fam. Med.* 2009, 22, 17-23. doi: 10.3122/jabfm.2009.01.080033.
172. Shargorodsky, J.; Curhan, S.G.; Curhan, G.C.; Eavey, R. Change in prevalence of hearing loss in US adolescents. *JAMA*. 2010, 304, 772-778. doi: 10.1001/jama.2010.1124.
173. Shimokura, R.; Soeta, Y. Listening level of music through headphones in train car noise environments. *J. Acoust. Soc. Am.* 2012, 132, 1407-1416. doi: 10.1121/1.4740472.
174. Sliwinska-Kowalska, M.; Davis, A. Noise-induced hearing loss. *Noise Health* 2012, 14, 274-280. doi: 10.4103/1463-1741.104893.
175. Smoorenburg, G.F. Risk of noise-induced hearing loss following exposure to Chinese firecrackers. *Audiology* 1993, 32, 333-343.
176. Stewart, M.; Meinke, D.K.; Snyders, J.K.; Howerton, K. Shooting habits of youth recreational firearm users. *Int. J. Audiol.* 2014, 53, 26-34. doi: 10.3109/14992027.2013.857437.
177. Suckfuell, M.; Canis, M.; Strieth, S.; Scherer, H.; Haisch, A. Intratympanic treatment of acute acoustic trauma with a cell-permeable JNK ligand: a prospective randomized phase I/II study. *Acta Otolaryngol.* 2007, 127, 938-942.
178. Sulkowski, W.J. Noise-induced hearing loss in children and youth: causes and prevention. *Med. Pr.* 2009, 60, 513-517.
179. Sumit, A.F.; Das, A.; Sharmin, Z.; Ahsan, N.; Ohgami, N.; Kato, M.; Akhand, A.A. Cigarette smoking causes hearing impairment among Bangladeshi population. *PLoS ONE.* 2015, 10, e0118960. doi: 10.1371/journal.pone.0118960.
180. Taljaard, D.S.; Leishman, N.F.; Eikelboom, R.H. Personal listening devices and the prevention of noise induced hearing loss in children: the Cheers for Ears Pilot Program. *Noise Health* 2013, 15, 261-268. doi: 10.4103/1463-1741.113523.
181. Tambs, K.; Hoffman, H.J.; Borchgrevink, H.M.; Holmen, J.; Samuelsen, S.O. Hearing loss induced by noise, ear infections, and head injuries: results from the Nord-Trondelag Hearing Loss Study. *Int. J. Audiol.* 2003, 42, 89-105.
182. Torre, P. Young adults' use and output level settings of personal music systems. *Ear Hear.* 2008, 29, 791-799. doi: 10.1097/AUD.0b013e31817e7409.
183. Torre, P.; Grace, J. Changes in distortion product otoacoustic emission components after music exposure. *J. Am. Acad. Audiol.* 2014, 25, 804-813. doi: 10.3766/jaaa.25.9.3.
184. Trzaskowski, B.; Jędrzejczak, W.W.; Piłka, E.; Cieślicka, M.; Skarżyński, H. Otoacoustic emissions before and after listening to music on a personal player. *Med. Sci. Monit.* 2014, 20, 1426-1431. doi: 10.12659/MSM.890747.
185. Tung, C.Y.; Chao, K.P. Effect of recreational noise exposure on hearing impairment among teenage students. *Res. Dev. Disabil.* 2013, 34, 126-132. doi: 10.1016/j.ridd.2012.07.015.

186. Twardella, D.; Perez Alvarez, C.; Steffens, T.; Fromme, H.; Raab, U. Hearing loss in adolescents due to leisure noise. The OHRKAN study. *Bundesgesundheitsbla.* 2011, 54, 965-971. doi: 10.1007/s00103-011-1321-2.
187. Ulrich, R.F.; Pinheiro, M.L. Temporary hearing losses in teen-agers attending repeated rock-and-roll sessions. *Acta. Otolaryngol.* 1974, 77, 51-55.
188. Vinay, S.N.; Moore, B.C. Effects of the use of personal music players on amplitude modulation detection and frequency discrimination. *J. Acoust. Soc. Am.* 2010, 128, 3634-3641. doi: 10.1121/1.3500679.
189. Vasconcellos, A.P.; Kyle, M.E.; Gilani, S.; Shin, J.J. Personally modifiable risk factors associated with pediatric hearing loss: a systematic review. *Otolaryngol. Head Neck Surg.* 2014, 15, 14-28.
190. Vogel, I.; Brug, J.; Hosli, E.J.; van der Ploeg, C.P.; Raat, H. MP3 players and hearing loss: adolescents' perceptions of loud music and hearing conservation. *J. Pediatr.* 2008, 152, 400-404. doi: 10.1016/j.jpeds.2007.07.009.
191. Vogel, I.; Brug, J.; van der Ploeg, C.P.; Raat, H. Adolescents risky MP3-player listening and its psychosocial correlates. *Health Educ. Res.* 2011, 26, 254-264.
192. Vogel, I.; Brug, J.; van der Ploeg, C.P.; Raat, H. Prevention of adolescents' music-induced hearing loss due to discotheque attendance: a Delphi study. *Health Educ. Res*. 2009, 24, 1043-1050. doi: 10.1093/her/cyp031.
193. Vogel, I.; Brug, J.; van der Ploeg, C.P.; Raat, H. Strategies for the prevention of MP3-induced hearing loss among adolescents: expert opinions from a Delphi study. *Pediatrics* 2009, 123, 1257-1262. doi: 10.1542/peds.2008-2291.
194. Vogel, I.; Brug, J.; van der Ploeg, C.P.; Raat, H. Young people's exposure to loud music: a summary of the literature. *Am. J. Prev. Med.* 2007, 33, 124-133.
195. Vogel, I.; Brug, J.; van der Ploeg, C.P.; Raat, H. Adolescents risky MP3-player listening and its psychosocial correlates. *Health Educ. Res.* 2011, 26, 254-264.
196. Vogel, I.; van de Looij-Jansen, P.M.; Mieloo, C.L.; Burdorf, A.; de Waart, F. Risky music-listening behaviors and associated health-risk behaviors. *Pediatrics* 2012, 129, 1097-1103. doi: 10.1542/peds.2011-1948.
197. Vogel, I.; Verschuure, H.; van der Ploeg, C.P.; Brug, J.; Raat, H. Adolescents and MP3 players: too many risks, too few precautions. *Pediatrics* 2009, 123, e953-958. doi: 10.1542/peds.2008-3179.
198. Vogel, I.; Verschuure, H.; van der Ploeg, C.P.; Brug, J.; Raat, H. Estimating adolescent risk for hearing loss based on data from a large school-based survey. *Am. J. Public Health* 2010, 100, 1095-1100.
199. Wash, P.; Dance, S. MP3 listening levels on London underground for music and speech. *Applied Acoustics.* 2013, 74, 850–855. DOI: 10.1016/j.apacoust.2012.12.008.
200. Weichbold, V.; Holzer, A.; Newesely, G.; Stephan, K. Results from high-frequency hearing screening in 14- to 15-year old adolescents and their relation to self-reported exposure to loud music. *Int. J. Audiol.* 2012, 51, 650-654. doi: 10.3109/14992027.2012.679747.
201. Weichbold, V.; Zorowka, P. Can a hearing education campaign for adolescents change their music listening behavior? *Int. J. Audiol.* 2007, 46, 128-133.
202. Weinreich, H.M.; Jabbour, N.; Levine, S.; Yueh, B. Limiting hazardous noise exposure from noisy toys: simple, sticky solutions. *Laryngoscope* 2013, 123, 2240-2244. doi: 10.1002/lary.23667.
203. Widén, S.E.; Erlandsson, S.I. Self-reported tinnitus and noise sensitivity among adolescents in Sweden. *Noise Health* 2004, 7, 29-40.
204. Widén, S.E.; Erlandsson, S.I. The influence of socio-economic status on adolescent attitude to social noise and hearing protection. *Noise Health* 2004, 7, 59-70.
205. Widén, S.E.; Holmes, A.E.; Erlandsson, S.I. Reported hearing protection use in young adults from Sweden and the USA: effects of attitude and gender. *Int. J. Audiol*. 2006, 45, 273-280.
206. Widén, S.E. A suggested model for decision-making regarding hearing conservation: towards a systems theory approach. *Int. J. Audiol.* 2013, 52, 57-64. doi: 10.3109/14992027.2012.728724.
207. Williams, W.; Purnell, J. The statistical distribution of expected noise level output from commonly available personal stereo players. *Acoust. Aust.* 2010, 38, 119-122.
208. Williams, W. Trends in listening to personal stereos. *Int. J. Audiol*. 2009, 48, 784-788.
209. Worthington, D.A.; Siegel, J.H.; Wilber, L.A.; Faber, B.M.; Dunckley, K.T.; Garstecki, D.C.; Dhar, S. Comparing two methods to measure preferred listening levels of personal listening devices. *J. Acoust. Soc. Am.* 2009, 125, 3733-3741. doi: 10.1121/1.3125798.
210. Xu, Z.; Li, Z.; Chen, Y.; He, Y.; Chunyu, X.; Wang, F.; Zhang, P.; Gao, L.; Qiu, S.; Liu, S.; Qiao, L.; Qiu, J. Hearing the impact of MP3 on a survey of middle school students. *J. Clin. Otorhinolaryngol. Head Neck Surg.* 2011, 25, 151-153.
211. Yaremchuk, K.; Dickson, L.; Burk, K.; Shivapuja, B.G. Noise level analysis of commercially available toys. *Int. J. Pediatr. Otorhinolaryngol.* 1997, 41, 187-197.
212. Yassi, A.; Pollock, N.; Tran, N.; Cheang, M. Risks to hearing from a rock concert. *Can. Fam. Physician*. 1993, 39, 1045-1050.
213. Zannin, P.H.; Calixto, A.; Diniz, F.B.; Ferreira, J.A.; Schuhli, R.B. Annoyance caused by urban noise to the citizens of Curitiba. *Brazil Rev. Saude Publica.* 2002, 36, 521-524.
214. Zardouz S, Shahriari SR, Djalilian HR. Noise-induced hearing loss from MP3 players. *Arch. Otolaryngol. Head Neck Surg.* 2010, 136, 1280. doi: 10.1001/archoto.2010.208. - author reply
215. Zeigler, M.C.; Taylor, J.A. The effects of Tinnitus awareness survey on college music majors' hearing conservation behaviors. *Med. Probl. Perform. Art*. 2001, 16, 136-143.
216. Zenner, H.P.; Struwe, V.; Schuschke, G.; Spreng, M.; Stange, G.; Plath, P.; Babisch, W.; Rebentisch, E.; Plinkert, P.; Bachmann, K.D.; Ising, H.; Lehnert, G. Hearing loss caused by leisure noise. *HNO*. 1999, 47, 236-248.
217. Zhai, S.; Jiang, S.; Gu, R.; Yang, W.; Wang, P. Effects of impulse noise on cortical response threshold and inner ear activity of succinic dehydrogenase and acetylcholinesterase in guinea pigs. *Acta. Otolaryngol.* 1998, 118, 813-816.

218. Zhao, F.; Manchaiah, V.K.; French, D.; Price, S.M. Music exposure and hearing disorders: an overview. *Int. J. Audiol.* 2010, 49, 54-64. doi: 10.3109/14992020903202520.
219. Zocoli, A.M.; Morata, T.C.; Marques, J.M.; Corteletti, L.J. Brazilian young adults and noise: attitudes, habits, and audiological characteristics. *Int. J. Audiol.* 2009, 48, 692-699.
220. [No authors listed]. Personal music listening devices and hearing loss. *Mayo Clin Health Lett*. 2010, 28, 4.

© 2017 by the authors. Submitted for possible open access publication under the terms and conditions of the Creative Commons Attribution (CC BY) license (http://creativecommons.org/licenses/by/4.0/).
